# Fecal bile acid dysmetabolism and reduced ursodeoxycholic acid correlate with novel microbial signatures in feline chronic kidney disease

**DOI:** 10.3389/fmicb.2024.1458090

**Published:** 2024-10-21

**Authors:** John C. Rowe, Stacie C. Summers, Jessica M. Quimby, Jenessa A. Winston

**Affiliations:** ^1^Department of Veterinary Clinical Sciences, The Ohio State University College of Veterinary Medicine, Columbus, OH, United States; ^2^Comparative Hepatobiliary Intestinal Research Program (CHIRP), The Ohio State University College of Veterinary Medicine, Columbus, OH, United States; ^3^Department of Clinical Sciences, Oregon State University Carlson College of Veterinary Medicine, Corvallis, OR, United States

**Keywords:** bile acid inducible (*bai*) operon, chronic kidney disease (CKD), 7α-dehydroxylation, *Peptacetobacter hiranonis*, *Oscillospirales*, ursodeoxycholic acid, gut microbiota, dysbiosis

## Abstract

**Background:**

Microbial-derived secondary bile acids (SBAs) are reabsorbed and sensed via host receptors modulating cellular inflammation and fibrosis. Feline chronic kidney disease (CKD) occurs with progressive renal inflammation and fibrosis, mirroring the disease pathophysiology of human CKD patients.

**Methods:**

Prospective cross-sectional study compared healthy cats (*n* = 6) with CKD (IRIS Stage 2 *n* = 17, Stage 3 or 4 *n* = 11). Single timepoint fecal samples from all cats underwent targeted bile acid metabolomics. 16S rRNA gene amplicon sequencing using DADA2 with SILVA taxonomy characterized the fecal microbiota.

**Results:**

CKD cats had significantly reduced fecal concentrations (median 12.8 ng/mg, Mann–Whitney *p* = 0.0127) of the SBA ursodeoxycholic acid (UDCA) compared to healthy cats (median 39.4 ng/mg). Bile acid dysmetabolism characterized by <50% SBAs was present in 8/28 CKD and 0/6 healthy cats. Beta diversity significantly differed between cats with <50% SBAs and > 50% SBAs (PERMANOVA *p* < 0.0001). Twenty-six amplicon sequence variants (ASVs) with >97% nucleotide identity to *Peptacetobacter hiranonis* were identified. *P. hiranonis* combined relative abundance was significantly reduced (median 2.1%) in CKD cats with <50% SBAs compared to CKD cats with >50% SBAs (median 13.9%, adjusted *p* = 0.0002) and healthy cats with >50% SBAs (median 15.5%, adjusted *p* = 0.0112). *P. hiranonis* combined relative abundance was significantly positively correlated with the SBAs deoxycholic acid (Spearman *r* = 0.5218, adjusted *p* = 0.0407) and lithocholic acid (Spearman *r* = 0.5615, adjusted *p* = 0.0156). Three *Oscillospirales* ASVs and a *Roseburia* ASV were also identified as significantly correlated with fecal SBAs.

**Clinical and translational importance:**

The gut-kidney axis mediated through microbial-derived SBAs appears relevant to the spontaneous animal CKD model of domestic cats. This includes reduced fecal concentrations of the microbial-derived SBA UDCA, known to regulate inflammation and fibrosis and be reno-protective. Microbes correlated with fecal SBAs include *bai* operon containing *P. hiranonis*, as well as members of *Oscillospirales*, which also harbor a functional *bai* operon. Ultimately, CKD cats represent a translational opportunity to study the role of SBAs in the gut-kidney axis, including the potential to identify novel microbial-directed therapeutics to mitigate CKD pathogenesis in veterinary patients and humans alike.

## 1 Introduction

Primary bile acids (PBAs) classically are lipid molecules produced and secreted by the liver into the intestinal tract to aid in digestion and absorption of nutrients such as fats (Ciaula et al., 2018). Beyond digestion, upon reabsorption bile acids also act as signaling molecules to orchestrate host physiology through bile acid activated receptors, including the nuclear farnesoid X receptor (FXR) and Takeda G protein-coupled 5 Receptor (TGR5) (Perino and Schoonjans, 2022). The composition of the bile acid pool available for intestinal reabsorption is dictated by the microbial community in the gut, or microbiota (Collins et al., 2023). The microbiota perform biotransformations of PBAs into microbial-derived secondary bile acids (SBAs) through enzymatic reactions that vastly expand the host-derived bile acid pool (Winston and Theriot, 2020). Microbial biotransformation potential is a product of the genes harbored by the gut microbiota, with only select microbes capable of performing important gatekeeping reactions to bile acid pool diversification (Collins et al., 2023; Winston and Theriot, 2020). For example, deconjugation of PBAs by gut microbes possessing bile salt hydrolase (BSH) makes unconjugated PBAs available for further biotransformation (Foley et al., 2019). Subsequent 7α-dehydroxylation performed by gut microbes containing the bile acid inducible (*bai*) operon allows for the conversion of the PBAs cholic acid (CA) and chenodeoxycholic acid (CDCA) into the SBAs deoxycholic acid (DCA) and lithocholic acid (LCA), respectively (Collins et al., 2023; Winston and Theriot, 2020; Funabashi et al., 2020). A limited number of culturable microbes are known to perform this function: *Clostridium scindens* (Kitahara et al., 2000), *Clostridium hylemonae* (Ridlon et al., 2010), *Peptacetobacter* *hiranonis* (formerly *Clostridium hiranonis*) (Kitahara et al., 2001), and *Extibacter muris* (in mice) (Streidl et al., 2021). However, recent metagenomic evidence suggests that currently unculturable members of the human gut microbiota within the order *Oscillospirales* may also harbor a functional *bai* operon (Vital et al., 2019; Kim et al., 2022). Separate reactions performed by microbes with hydroxysteroid dehydrogenases (HSDHs) allow for the generation of additional SBAs, such as ursodeoxycholic acid (UDCA) from CDCA (Collins et al., 2023; Winston and Theriot, 2020). As a result of microbial generation of SBAs, in healthy humans greater than 90% of fecal bile acids are SBAs (Ridlon et al., 2006). This composition is less clearly defined in healthy cats with most studies presenting healthy cat populations with a predominance of SBAs (Rowe and Winston, 2024), though isolated publications have included individuals with <50% SBAs within presumptively healthy cat populations (Sung et al., 2023).

Deviation of the gut microbiota from normal, known as dysbiosis, can alter microbial community function such that bile acid transformation is also impacted, creating a bile acid dysmetabolism. Bile acid dysmetabolism has been connected to multiple disease states in humans including *Clostridioides difficile* infection (Winston and Theriot, 2016), inflammatory bowel disease (Lloyd-Price et al., 2019), neurologic disease (McMillin and DeMorrow, 2016), cardiovascular disease (Rodríguez-Morató and Matthan, 2020), obesity, type 2 diabetes, dyslipidemia, and nonalcoholic fatty liver disease (Chávez-Talavera et al., 2017), as well as chronic kidney disease (CKD) (Chen et al., 2019; Wang et al., 2016; Li et al., 2019). Though less investigated, bile acid dysmetabolism is also recognized in various disease states of dogs and cats (Rowe and Winston, 2024).

Cats represent a spontaneous translational disease model of CKD, as development of CKD is reported to occur in up to 80% of cats 15 years or older (Bartges, 2012; Marino et al., 2014). CKD pathogenesis includes progressive tubulointerstitial inflammation and fibrosis (Jepson, 2016; McLeland et al., 2015). Given that the host bile acid activated receptors FXR and TGR5 are expressed in the kidney and have the potential to modulate host inflammatory and fibrotic responses, investigation of a bile acid dysmetabolism that may contribute to or mitigate CKD progression is warranted. Indeed, rodent models have demonstrated these mechanisms to be reno-protective in kidney injury induced via cisplatin (Yang et al., 2020) and gentamicin (Abd-Elhamid et al., 2018). To date, dysbiosis resulting in bile acid dysmetabolism in cats has been minimally explored (Rowe and Winston, 2024). Still, reduced alpha diversity in the gut microbial community has been shown in cats with CKD (Summers et al., 2019), and a single report investigating fecal metabolome in cats with CKD (*n* = 10) did not document a bile acid dysmetabolism (Hall et al., 2020). Further characterization of potential dysbiosis and resulting bile acid dysmetabolism in cats may provide insight into microbial directed therapeutic interventions for cats with CKD and microbial mechanisms that are conserved across species, including humans with CKD.

The aims of this study were (1) to determine if fecal bile acid dysmetabolism exists in cats with CKD, and (2) characterize gut microbiota features associated with the bile acid dysmetabolism in the context of feline CKD using targeted fecal bile acid quantification and 16S rRNA gene sequencing of fecal samples collected from cats with and without CKD.

## 2 Materials and methods

### 2.1 Study population and design

The present study utilized fecal samples collected from a prior study (Summers et al., 2019) and applied a post-hoc evaluation of fecal bile acid concentrations. Cats presenting to the Colorado State University Veterinary Teaching Hospital (CSU-VTH) were prospectively enrolled into a cross-sectional study approved by the CSU-VTH Institutional Animal Care and Use Committee (IACUC) as previously described (Summers et al., 2019). Briefly, enrollment was performed from August 2016 to August 2017 and yielded a population of cats with CKD (*n* = 28) and a healthy older control population (*n* = 6). All cats with CKD met inclusion following evaluation of client history, physical examination, medical record review, complete blood count, serum chemistry (including creatinine >1.6 mg/dL), urinalysis (including urine specific gravity (USG) < 1.035), serum total thyroxine concentration, blood pressure, fecal flotation, and urine protein to creatinine ratio (if 1+ protein or greater determined by urinalysis) (Summers et al., 2019). Cats with CKD were staged according to the International Renal Interest Society (IRIS) Guidelines (Stage 2 *n* = 17, Stage 3 or 4 *n* = 11) (IRIS Kidney, 2023). All healthy older control cats were owned by employees, veterinary students, or staff at CSU-VTH, were at least 8 years old, and underwent the same laboratory screening. Health status was determined by a veterinary board-certified internist based on clinical history, physical examination, available prior medical record review, and normal laboratory screening tests including serum creatinine <1.6 mg/dL and USG > 1.035 (Summers et al., 2019). Exclusion criteria included the administration of antimicrobials, antacids, or probiotics within 6 weeks of enrollment. Cats with uncontrolled hyperthyroidism as well as suspicion or confirmation of gastrointestinal disease, including gastrointestinal parasitism and food responsive enteropathy, were also excluded from the population (Summers et al., 2019). Diet information was collected from all owners; however, no dietary exclusion criteria were utilized. Basic demographic information is shown in Supplementary Table S1.

In the present study, healthy cats were required to have an ideal body condition score (BCS) of 4 or 5 out of 9 and did not include any cats with a BCS of 6 or greater, given that the gut microbiota and microbial-derived metabolites can be impacted by obesity status in both cats and people (Cline et al., 2021; Kieler et al., 2016; Rowe et al., 2024; Pinart et al., 2022). This restriction accounts for four less healthy older control cats compared to the prior study (Summers et al., 2019). A single CKD cat with an obese BCS (8/9) was also removed from the present analysis due to the possible confounding influence of comorbid obesity on the gut microbiota. CKD cats with overweight BCS (6 or 7 out of 9) were retained in the analysis as current guidelines recommend maintaining weight in CKD cats with these BCSs (Cline et al., 2021), thus capturing a realistic client-owned CKD cat population.

A fresh naturally voided fecal sample frozen within 24 h was used for microbiome and bile acid analyses. All fecal samples were collected by owners and placed on ice until being frozen at −80°C for further analysis. The prior study first utilized fecal samples for microbiota analysis (Summers et al., 2019). Targeted bile acid metabolomics were performed on remaining stored samples with sufficient fecal material. A single healthy cat and a single CKD cat from the prior study did not have sufficient sample for targeted bile acid metabolomics.

Within CKD cats, a subpopulation of 8/28 cats with <50% fecal SBAs were also analyzed in comparison to the healthy cats (*n* = 6) as well as CKD cats with >50% fecal SBAs (*n* = 20).

### 2.2 Quantification of fecal bile acid concentrations

Frozen fecal samples were shipped to and then analyzed by a fee for service laboratory (Metabolon Inc.) using liquid chromatography and tandem mass spectrometry (LC–MS/MS) to obtain fecal bile acid concentrations. This targeted metabolomic approach evaluated 15 fecal bile acids. The unconjugated bile acids assessed were cholic acid (CA), chenodeoxycholic acid (CDCA), deoxycholic acid (DCA), lithocholic acid (LCA), and ursodeoxycholic acid (UDCA). The taurine and glycine conjugated forms of all five unconjugated bile acids were also assessed. Briefly, calibration of all measured bile acids was performed with eight different known concentrations spiked into an acidified methanol solution. Quality control samples, calibration samples, and study samples were all also spiked with a labeled internal standard and subjected to protein precipitation with an organic solvent (acidified methanol). After centrifugation, organic supernatant was dried using a stream of nitrogen. Dried extracts were then reconstituted and injected onto an Agilent 1,290 Infinity/Sciex QTRAP 6500 LC–MS/MS system equipped with a C18 reverse phase ultra-high performance liquid chromatography (UHPLC) column. The mass spectrometer was operated in negative mode using electrospray ionization. Quantitation was performed using a weighted linear least squares regression generated from fortified calibration standards prepared immediately prior to each sample run. Raw data were collected and processed using AB SCIEX software Analyst (v1.6.3). Fecal bile acid concentrations are reported as nanogram per milligram of feces.

### 2.3 Fecal microbiota analysis

Publicly available forward-end read 16S rRNA gene amplicon sequencing data were obtained from the National Institutes of Health Sequence Read Archive from accession number SRP 117611, which were generated as previously described (Summers et al., 2019). Paired-end sequences were unavailable for evaluation; therefore, the publicly available forward-end reads were utilized. Python (v3.7.16) was used to obtain sequences using fasterq-dump (SRA Toolkit v3.0.5) (GitHub, 2023) and then forward primer sequence (5’-GTGCCAGCMGCCGCGGTAA-3′) was removed from all reads using cutadapt (v1.18) (Martin, 2011).

Sequence analysis of the V4 region of 16S rRNA gene amplicons was then performed through R Studio (v2023.03.1 + 446) (R Core Team, 2013). Further sequence trimming, filtering, and chimera removal was performed, including truncation of sequence length to 240 base pairs, and amplicon sequence variants (ASVs) generated using the DADA2 pipeline (v1.18.0) (Callahan et al., 2016). Taxonomy was then assigned to ASVs using the SILVA 16S rRNA sequence database (v138.1) (Quast et al., 2013). Subsequent taxonomy table creation was performed with the phyloseq package (v1.42.0) in R Studio (McMurdie and Holmes, 2013).

Evaluation of microbial community alpha and beta diversity measures utilized both phyloseq (v1.42.0) and vegan (v2.6–4) packages in R Studio (McMurdie and Holmes, 2013; Oksanen et al., 2022). Alpha diversity metrics that were evaluated included total number of observed ASVs, Shannon Diversity Index, and Inverse Simpson Diversity Index. Further visualization of alpha diversity metrics was generated using GraphPad Prism (Prism 10 v10.1.0 for macOS, GraphPad Software LLC, La Jolla, CA, USA). Beta diversity was assessed using Bray–Curtis dissimilarity distances and visualized via non-metric multidimensional scaling (NMDS) with a stress threshold of <0.2 considered acceptable and plots were generated using ggplot2 (v3.4.0) (Clarke, 1993).

Further evaluation of 16S rRNA gene amplicon sequence taxonomy was performed using National Center for Biotechnology Information (NCBI) Basic Local Alignment Search Tool (BLAST) (Altschul et al., 1990) as well as the NCBI Multiple Sequence Alignment Viewer (v1.25.0).

### 2.4 Statistical analysis

Within GraphPad Prism, data were assessed for normality with the Shapiro–Wilk test and determined to be non-normally distributed for both relative abundance and fecal bile acid concentration data. The non-parametric two-tailed Mann–Whitney and Kruskal-Wallis tests were applied as appropriate and significance determined following adjustment for false discovery rate (FDR). All FDR tests performed throughout this study used the Benjamini, Krieger, and Yekutieli method (Benjamini et al., 2006).

The alpha diversity metric of total observed ASVs were normally distributed, passing the Shapiro–Wilk test, and thus a parametric one-way ANOVA was applied for multiple comparisons. Differences in microbial community beta diversity were assessed by permutational multivariate analysis of variance (PERMANOVA) using the phyloseq (v1.42.0) and vegan (v2.6–4) packages in R Studio.

Differentially abundant taxa were identified using the DESeq2 package (v1.30.0) in R Studio (Love et al., 2014). Parameters of the test were set to Test = Wald, FitType = Parametric, Cook’s Cutoff = FALSE, independentFiltering = FALSE (i.e., not applied to the dataset), and Benjamini-Hochberg post-hoc correction was applied to generate false discovery rate adjusted *p* values similar to previously described (Nealon et al., 2023). Comparisons were made on a log2 fold change (log2FC) basis and were visualized using the package EnhancedVolcano (v1.16.0) in R Studio.

MetaboAnalyst (v5.0) was used to analyze fecal bile acid concentration data. No additional transformation, scaling, or normalization was performed on concentration data prior to analysis. Analyses performed within MetaboAnalyst included principal component analysis (prcomp package and R script chemometrics.R) and Random Forest machine learning algorithm (randomForest package). All package versions were contained within MetaboAnalyst v5.0 using R (v4.2.2). Additional fecal bile acid concentration heatmap generation was performed in R Studio with the heatmap.2 function from the gplots package (v3.1.3) and hierarchical clustering performed with the hclust function.

Two-tailed Spearman rank correlation using GraphPad Prism and R Studio was used to assess correlations between the relative abundances of taxa and concentration of bile acids detected in fecal samples. Within R Studio, the psych (v2.2.9) package was utilized to first perform Spearman correlation analysis with the corr.test function, then the corrplot package (v0.92) was utilized to visualize the Spearman correlation matrices with the corrplot function.

## 3 Results

### 3.1 Fecal ursodeoxycholic acid is reduced in CKD cats

**Figure 1:**
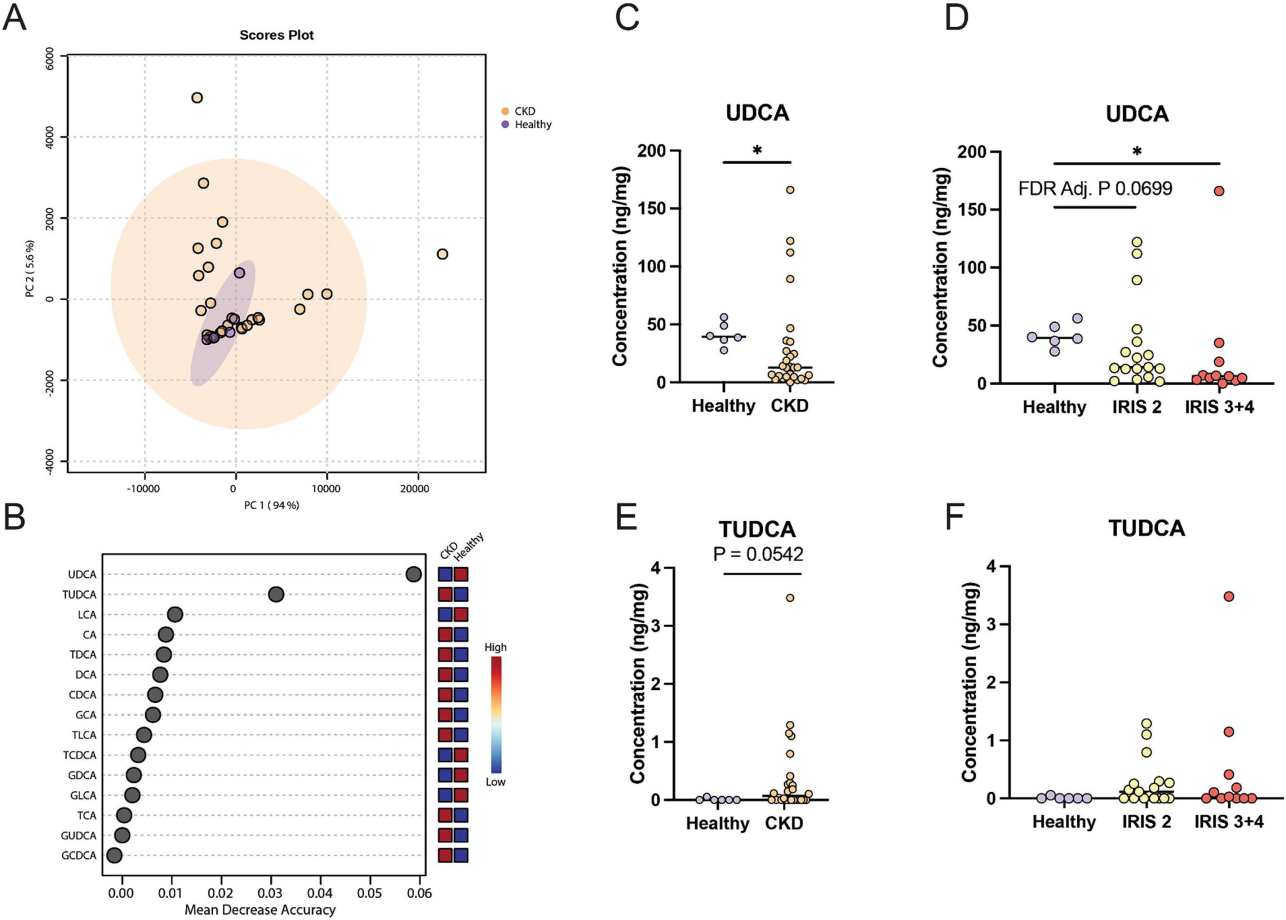
Fecal bile acid concentrations in cats with chronic kidney disease. **(A)** Principal component analysis of fecal bile acid concentrations in healthy cats (individual samples represented by purple points) and cats with CKD (individual samples represented by orange points) and a 95% confidence interval clouds for the clustering of each group. Variance explained by each principal component is listed on the corresponding axis. **(B)** Random Forest machine learning algorithm with dots corresponding to a fecal bile acid along the left-hand y-axis and the listed mean decrease in accuracy along the x-axis. Boxes along the right-hand y-axis demonstrate whether the fecal bile acid is high (red) or low (blue) in healthy and CKD cat groups. **(C)** Fecal concentration of UDCA in healthy cats and cats with CKD, significance determined via Mann–Whitney; * *p* < 0.05. **(D)** Fecal concentration of UDCA in healthy cats, cats with IRIS CKD Stage 2, and cats with IRIS CKD Stage 3 or 4, significance determined via Kruskal-Wallis with FDR adjustment; significant discoveries noted by *. **(E)** Fecal concentration of TUDCA in healthy cats and cats with CKD, significance determined via Mann–Whitney. **(F)** Fecal concentration of UDCA in healthy cats, cats with IRIS CKD Stage 2, and cats with IRIS CKD Stage 3 or 4 cats, significance determined via Kruskal-Wallis with FDR adjustment.

The profiles of fecal bile acid concentrations from healthy cats and cats with CKD were largely similar and demonstrated considerable overlap via principal component analysis (PCA) (Figure 1A). A Random Forest machine learning algorithm was used to assess which fecal bile acid concentrations may be able to best discriminate between healthy cats and cats with CKD (Figure 1B). Of the 15 fecal bile acids, TUDCA and UDCA best discriminated between healthy cats and cats with CKD. UDCA was in significantly greater concentration in feces of healthy cats (median 39.4 ng/mg) than cats with CKD (median 12.8 ng/mg, Mann–Whitney *p* = 0.0127) (Figure 1C). When subdivided by IRIS stage and compared to healthy cats, cats with IRIS Stage 2 CKD had reduced fecal UDCA (median 14.1 ng/mg, FDR adjusted *p* = 0.0699) and cats with IRIS Stage 3 or 4 CKD had significantly reduced fecal UDCA (median 6.2 ng/mg, FDR adj. *p* = 0.0092) (Figure 1D). There were no significant differences when TUDCA was assessed, though TUDCA was only detected in feces from one healthy cat (Figure 1E). In cats with CKD there was a trend of increasing fecal TUDCA concentration with IRIS stage 2 cats (median 0.115 ng/mg, FDR adj. *p* = 0.1621) and cats with IRIS Stage 3 or 4 (median 0.0279 ng/mg, FDR adj. *p* = 0.1621) compared to healthy cats (Figure 1F).

### 3.2 Gut microbiota members correlate with reduced fecal UDCA in CKD cats

**Figure 2:**
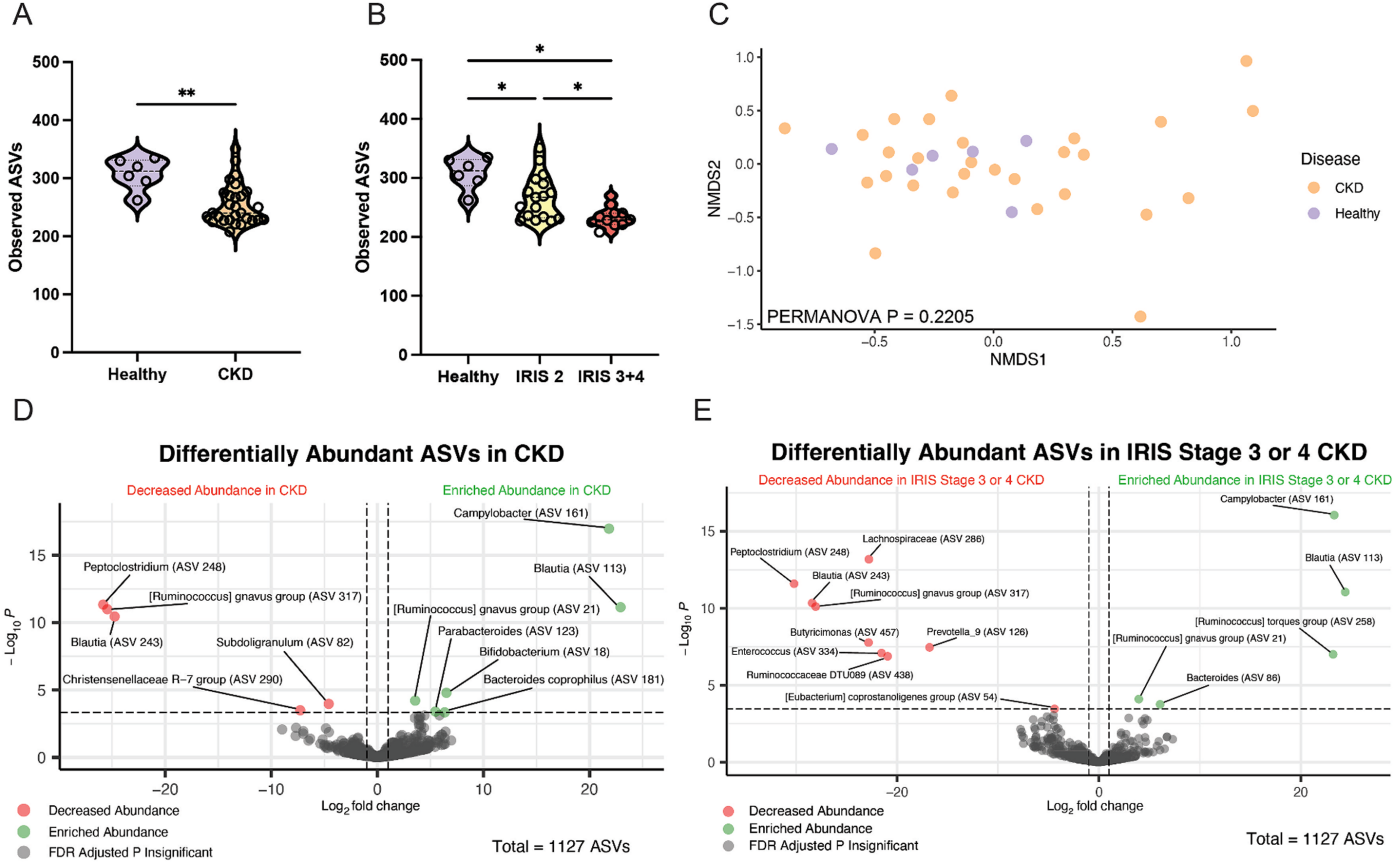
Fecal microbiota of cats with chronic kidney disease. **(A)** Alpha diversity in healthy cats and cats with CKD represented by violin plots of total observed ASVs and significance determined via Mann–Whitney; ** *p* < 0.01. **(B)** Alpha diversity in healthy cats, cats with IRIS CKD Stage 2, and cats with IRIS CKD Stage 3 or 4 cats represented by violin plots of total observed ASVs and significance determined via parametric one-way ANOVA with FDR adjustment; significant discoveries noted by *. **(C)** Beta diversity determined with Bray–Curtis dissimilarity distances depicted with non-metric multi-dimensional scaling (NMDS) plot with individual samples represented by points for healthy cats (purple) and cats with CKD (orange) and significance determined with PERMANOVA. **(D)** Volcano plot displaying differentially abundant ASVs determined with DESeq2 that are either reduced in abundance (red) or enriched in abundance (green) in cats with CKD compared to healthy cats. Points maintaining significance following FDR adjustment (*p* < 0.05) and having a log2FC of at least magnitude 2 are plotted and colored. All gray points do not meet these criteria. **(E)** Volcano plot displaying differentially abundant ASVs determined with DESeq2 that are either reduced in abundance (red) or enriched in abundance (green) in cats with IRIS CKD Stage 3 or 4 s compared to healthy. Points maintaining significance following FDR adjustment (*p* < 0.05) and having a log2FC of at least magnitude 2 are plotted and colored. All gray points do not meet these criteria.

Alpha diversity was significantly reduced in cats with CKD characterized by a reduced number of total observed ASVs in cats with CKD (median 240 ASVs, Mann–Whitney *p* = 0.0029) compared to healthy cats (median 312 ASVs) (Figure 2A). When IRIS stage was considered, both IRIS Stage 2 cats (median 267 ASVs, FDR adjusted *p* = 0.0105) and IRIS Stage 3 or 4 cats (median 229 ASVs, FDR adjusted *p* = 0.0001) had significantly fewer ASVs compared to healthy cats (Figure 2B). Moreover, IRIS Stage 3 or 4 cats had significantly fewer ASVs than cats with IRIS Stage 2 (FDR adjusted *p* = 0.0105) (Figure 2B). The beta diversity assessed by Bray-Curtis distances were not different between healthy cats and cats with CKD (PERMANOVA *p* = 0.2205) (Figure 2C). These findings are in agreement with the original analysis of this publicly available 16S rRNA gene amplicon sequencing data set, which utilized a different bioinformatics pipeline and taxonomy assignment database (Summers et al., 2019).

Differentially abundant ASVs were determined by log2 fold change (log2FC) with DESeq2 when all cats with CKD were compared to healthy cats (Figure 2D). An ASV belonging to the enteropathogen genus *Campylobacter* (ASV 161) was significantly enriched in cats with CKD (log2FC = 21.8, FDR adj. *p* < 0.0001). Separately, a *Peptoclostridium* sp. (ASV 248) was significantly reduced in abundance in cats with CKD (log2FC = −25.8, FDR adj. p < 0.0001). This ASV was further investigated with NCBI Blast and found to have 99.58% sequence identity with the 16S rRNA gene DNA sequence of *Peptacetobacter hiranonis* (Table 1).

**Table 1:** ASVs identified in cats with at least 97% sequence identity with *P. hiranonis* of the sequenced V4 region of the 16S rRNA gene.

ASV ID	Range % Relative Abundance	V4 region of 16S rRNA gene amplicon sequence 5′-3′	Silva taxonomy (v138.1)	NCBI BLAST Result(s)	Nucleotide Identity (%)
1	0.20–31.33%	TACGTAGGGGGCTAGCGTTATCCGGATTTACTGGGCGTAAAGGGTGCGTAGGCGGTCTTTCAAGTCAGGAGTTAAAGGCTACGGCTCAACCGTAGTAAGCTCCTGATACTGTCTGACTTGAGTGCAG GAGAGGAAAGCGGAATTCCCAGTGTAGCGGTGAAATGCGTAGATATTGGGAGGAACACCAGTAGCGAAGGCGGCTTTCTGGACTGTAACTGACGCTGAGGCACGAAAGCGTGG	Genus: *Peptoclostridium*	*P. hiranonis*	100%
74	0–4.05%	TACGTAGGGGGCTAGCGTTATCCGGATTTACTGGGCGTAAAGGG TGCGTAGGCGGTCTTTCAAGTCAGGAGTTAAAGGCTACGGCTCAACCGTAGTAAGCTCCTGATACTGTCTGACTTGAGTGCAGGAGAGGAA AGCGGAATTCCCAGTGTAGCGGTGAAATGCGTAGATATTGGGAGGAACATCAGTAGCGAAGGCGGCTTTCTGGACTGTAACTGACGCTGAGGCACGAAAGCGTGG	Genus: *Peptoclostridium*	Uncultured *P. hiranonis*	100% 99.58%
94	0–3.11%	TACGTAGGGGGCTAGCGTTATCCGGATTTACTGGGCGTAAAGGGTGCGTAGGCGGTCTTTCAAGTCAGGAGTTAAAGGCTACGGCTCAACCGTAGTAAGCTCCTGATACTGTCTGACTTGAGTGCAGGAGAGGAAAGTGGAATTCCCAGTGTAGCGGTGAAATGCGTAGATATTGGGAGGAACACCAGTAGCGAAGGCGGCTTTCTGGACTGTAACTGACGCTGAGGCACGAAAGCGTGG	Genus: *Peptoclostridium*	*C. hiranonis* *P. hiranonis*	100% 99.58%
115	0–1.59%	TACGTAGGGGGCTAGCGTTATCCGGATTTACTGGGCGTAAAGGGTGCGTAGGTGGTCTTTCAAGTCAGGAGTTAAAGGCTACGGCTCAACCGTAGTAAGCTCCTGATACTGTCTGACTTGAGTGCAGGAGAGGAAAGCGGAATTCCCAGTGTAGCGGTGAAATGCGTAGATATTGGGAGGAACACCAGTAGCGAAGGCGGCTTTCTGGACTGTAACTGACGCTGAGGCACGAAAGCGTGG	Genus: *Peptoclostridium*	*P. hiranonis*	99.58%
137	0–4.12%	TACGTAGGGGGCTAGCGTTATCCGGATTTACTGGGCGTAAAGGGTGC GTAGGCGGTCTTTCAAGTCAGGAGTTAAAGGCTACGGCTCAACCGTAGTAAGCTCCTGATACTATCTGACTTGAGTGCAGGAGAGGAAAGCGGAATTCCCAGTGTAGCGGTGAAATGCGTAGATATTGGGAGGAACACCAGTAGCGAAGGCGGCTTTCTGGACTGTAACTGACGCTGAGGCACGAAAGCGTGG	Genus: *Peptoclostridium*	*P. hiranonis*	99.58%
165	0–2.71%	TACGTAGGGGGTTAGCGTTATCCGGATTTACTGGGCGTAAAGGGTGCGTAGGCGGTCTTTCAAGTCAGGAGTTAAAGGCTACGGCTCAACCGTAGTAAGCTCCTGATACTGTCTGACTTGAGTGCAGGAGAGGAAAGCGGAATTCCCAGTGTAGCGGTGAAATGCGTAGATATTGGGAGGAACACCAGTAGCGAAGGCGGCTTTCTGGACTGTAACTGACGCTGAGGCACGAAAGCGTGG	Genus: *Peptoclostridium*	*P. hiranonis*	99.58%
176	0–1.70%	TACGTAGGGGGCTAGCGTTATCCGGATTTACTGGGCGTAAAGGGTGCGT AGGCGGTCTTTCAAGTCAGGAGTTAAAGGCTACGGCTCAACCGTAGTAAG CTCCTGATACTGTCTGACTTGAGTGCAGGAGAGGAAAGCGGAATTCCCAG TGTAGCGGTGAAATGCGTAGATATTGGGAGGAACACCAGTAGCGAAGGCGGTTTTCTGGACTGTAACTGACGCTGAGGCACGAAAGCGTGG	Genus: *Peptoclostridium*	*P. hiranonis*	99.58%
226	0–1.51%	TACGTAGGGGGCTAGCGTTATCCGGATTTACTGGGCGTAAAGGGTGCGTA GGCGGTCTTTCAAGTCAGGAGTTAAAGGCTACGGCTCAACCGTAGTAAGC TCCTGATACTGTCTGACTTGAGTGCAGGAGAGGAAAGCGGAATTCCCAGT GTAGTGGTGAAATGCGTAGATATTGGGAGGAACACCAGTAGCGAAGGCGGCTTTCTGGACTGTAACTGACGCTGAGGCACGAAAGCGTGG	Genus: *Peptoclostridium*	Uncultured *P. hiranonis*	100% 99.58%
236	0–1.34%	TACGTAGGGGGCTAGCGTTATCCGGATTTACTGGGCGTAAAGGGTGCGTA GGCAGTCTTTCAAGTCAGGAGTTAAAGGCTACGGCTCAACCGTAGTAAGC TCCTGATACTGTCTGACTTGAGTGCAGGAGAGGAAAGCGGAATTCCCAGT GTAGCGGTGAAATGCGTAGATATTGGGAGGAACACCAGTAGCGAAGGCGGCTTTCTGGACTGTAACTGACGCTGAGGCACGAAAGCGTGG	Genus: *Peptoclostridium*	*P. hiranonis*	99.58%
248	0–0.67%	TACGTAGGGGGCTAGCGTTATCCGGATTTACTGGGCGTAAAGGGTGCGT AGGCGGTCTTTCAAGTCAGGAGTTAAAGGCTACGGCTCAACCGTAGTAAG CTCCTGATACTGTCTGACTTGAGTGTAGGAGAGGAAAGCGGAATTCCCAG TGTAGCGGTGAAATGCGTAGATATTGGGAGGAACACCAGTAGCGAAGGCGGCTTTCTGGACTGTAACTGACGCTGAGGCACGAAAGCGTGG	Genus: *Peptoclostridium*	*P. hiranonis*	99.58%
256	0–1.15%	TACGTAGGGGGCTAGCGTTATCCGGATTTACTGGGCGTAAAGGGTGCGTA GGTGGTCTTTCAAGTCAGGAGTTAAAGGCTACGGCTCAACCGTAGTAAGC TCCTGATACTGTCTGACTTGAGTGCAGGAGAGGAAAGCGGAATTCCCAGT GTAGTGGTGAAATGCGTAGATATTGGGAGGAACACCAGTAGCGAAGGCGGCTTTCTGGACTGTAACTGACGCTGAGGCACGAAAGCGTGG	Genus: *Peptoclostridium*	Uncultured *P. hiranonis*	99.58% 99.17%
265	0–1.06%	TACGTAGGGGGCTAGCGTTATCCGGATTTACTGGGCGTAAAGGGTGCGTA GGCGGTCTCTCAAGTCAGGAGTTAAAGGCTACGGCTCAACCGTAGTAAGC TCCTGATACTGTCTGACTTGAGTGCAGGAGAGGAAAGCGGAATTCCCAGT GTAGCGGTGAAATGCGTAGATATTGGGAGGAACACCAGTAGCGAAGGCGGCTTTCTGGACTGTAACTGACGCTGAGGCACGAAAGCGTGG	Genus: *Peptoclostridium*	*P. hiranonis*	99.58%
297	0–0.77%	TACGTAGGGGGCTAGCGTTATCCGGATTTACTGGGCGTAAAGGGTGCGTA GGCGGTCTTTCAAGTCAGGAGTTAAAGACTACGGCTCAACCGTAGTAAGCTCCTGATACTGTCTGACTTGAGTGCAGGAGAGGAAAGCGGAATTCCCAGT GTAGCGGTGAAATGCGTAGATATTGGGAGGAACACCAGTAGCGAAGGCGGCTTTCTGGACTGTAACTGACGCTGAGGCACGAAAGCGTGG	Genus: *Peptoclostridium*	Uncultured *P. hiranonis*	100% 99.58%
308	0–0.43%	TACGTAGGGGGCTAGCGTTATCCGGATTTACTGGGCGTAAAGGGTGCGTAGGCGGTCTTTCAAGTCAGGAGTTAAAGGCTACGGCTCAACTGTAGTAAGCTCCTGATACTGTCTGACTTGAGTGCAGGAGAGGAAAGCGGAATTCCCAGTGTAGCGGTGAAATGCGTAGATATTGGGAGGAACACCAGTAGCGAAGGCGGCTTTCTGGACTGTAACTGACGCTGAGGCACGAAAGCGTGG	Genus: *Peptoclostridium*	Uncultured *P. hiranonis*	100% 99.58%
323	0–0.62%	TACGTAGGGGGCTAGCGTTATCCGGATTTACTGGGCGTAAAGGGTGCGTAGGCGGTCTTTCAAGTCAGGAGTTAAAGGCTACGGCTCAACCGTAGTAAGCTCCTGGTACTGTCTGACTTGAGTGCAGGAGAGGAAAGCGGAATTCCCAGTGTAGCGGTGAAATGCGTAGATATTGGGAGGAACACCAGTAGCAAAGGCGGCTTTCTGGACTGTAACTGACGCTGAGGCACGAAAGCGTGG	Genus: *Peptoclostridium*	*P. hiranonis*	99.17%
325	0–0.61%	TACGTAGGGGGCTAGCGTTATCCGGATTTACTGGGCGTAAAGGGTGCGTAGGCGGTCTTTCAAGTCAGGAGTTAAAGGCTACGGCTCAACCGTAGTAAGCTCCTGATACTGTCTGACTTGAGTGCAGGAGAGGAAAACGGAATTCCCAGTGTAGCGGTGAAATGCGTAGATATTGGGAGGAACACCAGTAGCGAAGGCGGCTTTCTGGACTGTAACTGACGCTGAGGCACGAAAGCGTGG	Genus: *Peptoclostridium*	Uncultured *P. hiranonis*	100% 99.58%
342	0–0.56%	TACGTAGGGGGCTAGCGTTATCCGGATTTACTGGGCGTAAAGGGTGCGTAGGCGGTCTTTCAAGTCAGGAGTTAAAGGCTACGGCTCAACCGTAGTAAGCTCCTGATACTGTCTGACTTGAGTGCAGGAGAGGAAAGCGGAATTCCCAGTGTAGCGGTGAAATGCGTAGATATTGGGAGGAACACCAGTAGCGAAGGCGGCTTTCTGGACTGTGACTGACGCTGAGGCACGAAAGCGTGG	Genus: *Peptoclostridium*	*P. hiranonis*	99.58%
374	0–0.49%	TACGTAGGGGGCTAGCGTTATCCGGATTTACTGGGCGTAAAGGGTGCGTAGGCGGTCTTTCAAGTCAGGAGTTAAAGGCTACGGCTCAACCGTAGTAAGCTCCTGATACTGTCTGACTTGAGTGCAGGAGAGGAAAGCGGAATTCCCAGTGTAGCGGTGAAATGCGTAGATATTGGGAGGAACACTAGTAGCGAAGGCGGCTTTCTGGACTGTAACTGACGCTGAGGCACGAAAGCGTGG	Genus: *Peptoclostridium*	Uncultured *P. hiranonis*	100% 99.58%
392	0–0.43%	TACGTAGGGGGCTAGCGTTATCCGGATTTACTGGGCGTAAAGGGTGCGTAGGCGGTCTTTCAAGTCAGGAGTTAAAGGCTACGGCTTAACCGTAGTAAGCTCCTGATACTGTCTGACTTGAGTGCAGGAGAGGAAAGCGGAATTCCCAGTGTAGCGGTGAAATGCGTAGATATTGGGAGGAACACCAGTAGCGAAGGCGGCTTTCTGGACTGTAACTGACGCTGAGGCACGAAAGCGTGG	Genus: *Peptoclostridium*	Uncultured *P. hiranonis*	100% 99.58%
393	0–0.43%	TACGTAGGGGGCTAGCGTTATCCGGATTTACTGGGCGTAAAGGGTGCGTAGGCGGTCTTTCAAGTCAGGAGTTAAAGGCTACGGCTCAACCGTAGTAAGCTCCTGATACTGTCTGACTTGAGTTCAGGAGAGGAAAGCGGAATTCCCAGTGTAGCGGTGAAATGCGTAGATATTGGGAGGAACACCAGTAGCGAAGGCGGCTTTCTGGACTGTAACTGACGCTGAGGCACGAAAGCGTGG	Genus: *Peptoclostridium*	*P. hiranonis*	99.58%
408	0–0.40%	TACGTAGGGGGCTAGCGTTATCCGGATTTACTGGGCGTAAAGGGTGCGTAGGCGGTCTTTCAAGTCAGGAGTTAAAGGCTACGGCTCAACCGTAGTAAGCTCCTGATACTGTCTGACTTGAGTGCAGGAGAGGAAAGCGGAATTCCCAGTGTAGCGGTGAAATGCGTAGATATTGGGAGGAATACCAGTAGCGAAGGCGGCTTTCTGGACTGTAACTGACGCTGAGGCACGAAAGCGTGG	Genus: *Peptoclostridium*	Uncultured *P. hiranonis*	100% 99.58%
442	0–0.04%	TACGTAGGGGGCTAGCGTTATCCGGATTTACTGGGCGTAAAGGGTGCGTAGGCGGTCTTTCAAGTCAGGAGTTAAAGGCTACGGCTCAACCGTAGTAAGCTCCTGATACTGTCTGACTTGAGTGCAGGAGAGGAAAGCGGAATTCCCAGTGTAGCGGTGAAATGCGTAGATATTGGGAGGAACACCAGTAGCGAAGGCGGCTTTCTGGACTGTAACTGACGCTGAGGCACGAAAGCTGGG	Genus: *Peptoclostridium*	*P. hiranonis*	99.58%
461	0–0.29%	TACGTAGGGGGCTAGCGTTATCCGGATTTACTGGGCGTAAAGGGTGCGTAGGCGGTCTTTCAAGTCAGGAGTTAAAGGCTACAGCTCAACCGTAGTAAGCTCCTGATACTGTCTGACTTGAGTGCAGGAGAGGAAAGCGGAATTCCCAGTGTAGCGGTGAAATGCGTAGATATTGGGAGGAACACCAGTAGCGAAGGCGGCTTTCTGGACTGTAACTGACGCTGAGGCACGAAAGCGTGG	Genus: *Peptoclostridium*	*P. hiranonis*	99.58%
495	0–0.25%	TACGTAGGGGGCTAGCGTTATCCGGATTTACTGGGCGTAAAGGGTGCGTAGGCGGTCTTTCAAGTCAAGAGTTAAAGGCTACGGCTCAACCGTAGTAAGCTCCTGATACTGTCTGACTTGAGTGCAGGAGAGGAAAGCGGAATTCCCAGTGTAGCGGTGAAATGCGTAGATATTGGGAGGAACACCAGTAGCGAAGGCGGCTTTCTGGACTGTAACTGACGCTGAGGCACGAAAGCGTGG	Genus: *Peptoclostridium*	*P. hiranonis*	99.58%
531	0–0.22%	TACGTAGGGGGCTAGCGTTATCCGGATTTACTGGGCGTAAAGGGTGCGTAGGCGGTCTTTCAAGTCAGGAGTTAAAGGCTACGGCTCAACCGTAGTAAGCTCCTGATACTGTTTGACTTGAGTGCAGGAGAGGAAAGTGGAATTCCCAGTGTAGCGGTGAAATGCGTAGATATTGGGAGGAACACCAGTAGCGAAGGCGGCTTTCTGGACTGTAACTGACGCTGAGGCACGAAAGCGTGG	Genus: *Peptoclostridium*	*C. hiranonis* *P. hiranonis*	99.58% 99.17%
558	0–0.06%	TACGTAGGGGGCTAGCGTTATCCGGATTTACTGGGCGTAAAGGGTGCGTAGGCGGTCTTTCAAGTCAGGAGTTAAAGGCTACGGCTCAACCGTAGTAAGCTCCTGATACTGTCTGACTTGAGTGCAGGAGAGGAAAGCGGAATTCCCAGTGTAGCGGTGAAATGCGTAGATATTAGGAGGAACATCAGTGGCGAAGGCGGCTTACTGGACTGAAACTGACACTGAGGCACGAAAGCGTGG	Family: Peptostreptococcaceae	Uncultured *P. hiranonis*	97.92% 97.50%
589	0–0.16%	TACGTAGGGGGCTAGCGTTATCCGGATTTACTGGGCGTAAAGGGTGCGTAGGCGGTCTTTCAAGTCAGGAGTTAAAGGCTACGGCTCAACCGTAGTAAGCTCCTGATACTGTCTGACTTGAGTGCAGGAGAGGAAAGCGGAATTCCCAGTGTAGCGGTGAAATGCGTAGATATTGGGAGGAACACCAGTGGCGAAGGCGGCTTTCTGGACTGTAACTGACGCTGAGGCACGAAAGCGTGG	Genus: *Peptoclostridium*	Uncultured *P. hiranonis*	100% 99.58%
707	0–0.08%	TACGTAGGGGGCTAGCGTTATCCGGATTTACTGGGCGTAAAGGGTGCGTAGGTGGTCTTTCAAGTCAGGAGTTAAAGGCTACGGCTCAACCGTAGTAAGCTCCTGATACTGTCTGACTTGAGTGCAGGAGAGGAAAGTGGAATTCCCAGTGTAGCGGTGAAATGCGTAGATATTGGGAGGAACACCAGTAGCGAAGGCGGCTTTCTGGACTGTAACTGACGCTGAGGCACGAAAGCGTGG	Genus: *Peptoclostridium*	*C. hiranonis* *P. hiranonis*	99.58% 99.17%

ASVs differentially abundant in only cats with IRIS Stage 3 or 4 CKD compared to healthy cats were also explored to capture microbial alterations that may exist with CKD progression (Figure 2E). Again, the same *Campylobacter* sp. (ASV 161) was enriched in cats with IRIS Stage 3 or 4 CKD (log2FC = 23.3, FDA adj. *p* < 0.0001). Similarly, the same *Peptoclostridium* sp. (ASV 248) was significantly reduced in abundance in cats with IRIS Stage 3 or 4 CKD (log2FC = −30.2, FDR adj. p < 0.0001). A microbe identified to the family level as *Lachnospiraceae* (ASV 286) was also significantly reduced in cats with IRIS Stage 3 or 4 CKD (log2FC = −22.8, FDR adj. *p* < 0.0001).

**Figure 3:**
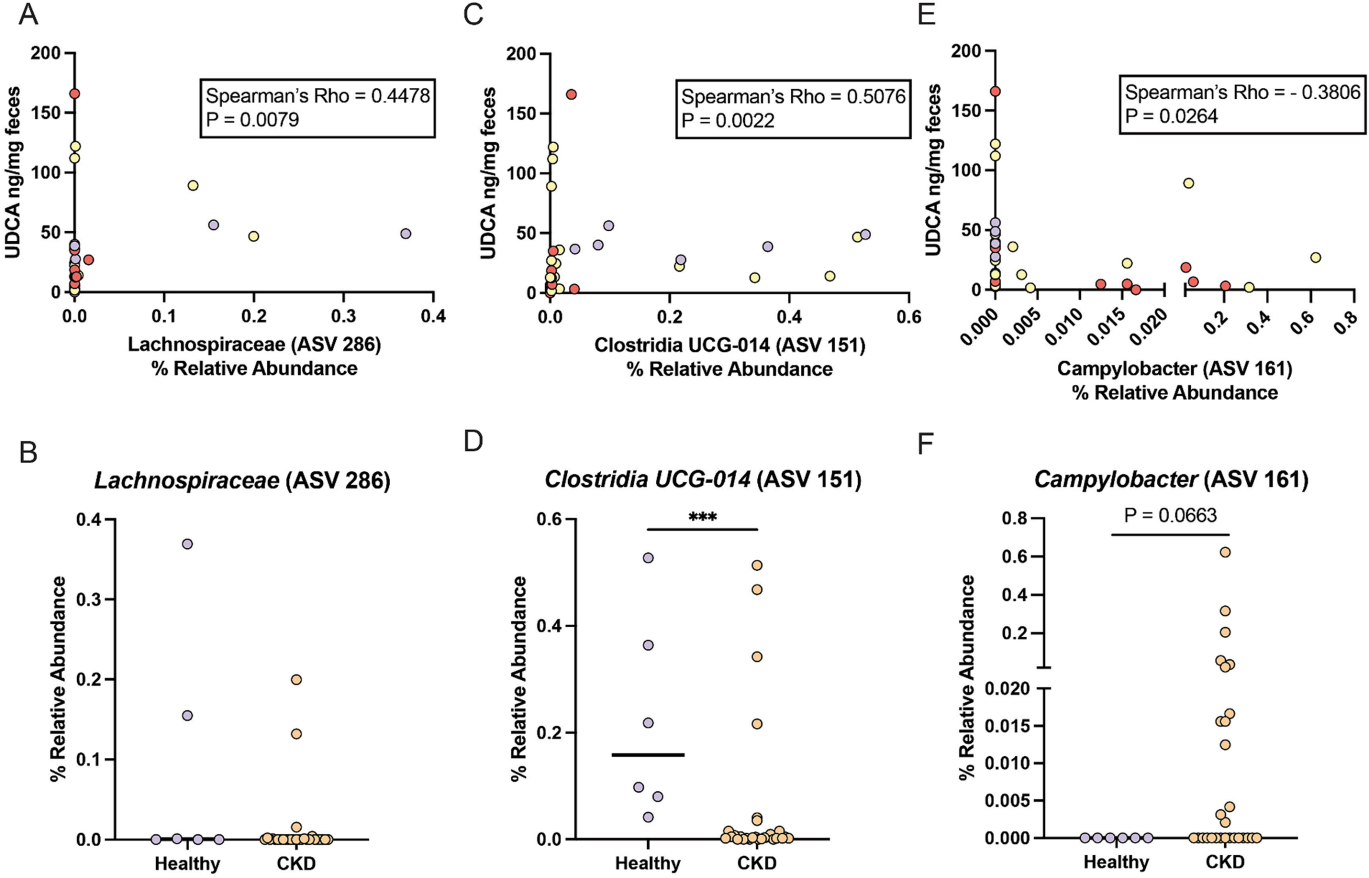
ASVs that correlate with reduced fecal UDCA concentrations in cats with CKD and healthy cats. **(A)** Spearman correlation of the relative abundance of *Lachnospiraceae* (ASV 286) and fecal UDCA concentration. Points are colored by healthy cats (purple), cats with IRIS CKD Stage 2 (yellow) and cats with IRIS CKD Stage 3 or 4 (red). **(B)** Relative abundance of *Lachnospiraceae* (ASV 286) in healthy cats (purple) and cats with CKD (orange) with significance determined by Mann–Whitney. **(C)** Spearman correlation of the relative abundance of *Clostridia UCG-014* (ASV 151) and fecal UDCA concentration. Points are colored by healthy cats (purple), cats with IRIS CKD Stage 2 (yellow) and cats with IRIS CKD Stage 3 or 4 (red). **(D)** Relative abundance of *Clostridia UCG-014* (ASV 151) in healthy cats (purple) and cats with CKD (orange) with significance determined by Mann–Whitney; *** *p* < 0.001. **(E)** Spearman correlation of the relative abundance of *Campylobacter* (ASV 161) and fecal UDCA concentration. Points are colored by healthy cats (purple), cats with IRIS CKD Stage 2 (yellow) and cats with IRIS CKD Stage 3 or 4 (red). **(F)** Relative abundance of *Campylobacter* (ASV 161) in healthy cats (purple) and cats with CKD (orange) with significance determined by Mann–Whitney.

*Lachnospiraceae* (ASV 286) had a significant moderate positive correlation with fecal UDCA concentration (Spearman *ρ* = 0.4478, *p* = 0.0079) (Figure 3A). *Lachnospiraceae* (ASV 286) was detected in 3/6 healthy cats (median relative abundance = 0.0005%; range = 0–0.37%) and 7/28 CKD cats (median relative abundance = 0%; range = 0–0.20%) (Figure 3B). An uncultured *Clostridia UCG-014* (ASV 151) not identified in the DESeq2 analysis had a stronger significant moderate positive correlation with fecal UDCA concentration (Spearman *ρ* = 0.5076, *p* = 0.0022) (Figure 3C). This *Clostridia UCG-014* (ASV 151) was detected in all healthy cats at a significantly greater relative abundance (median relative abundance = 0.16%; range = 0.04–0.53%) than in cats with CKD where it was detected in 22/28 cats (median relative abundance = 0.004%; range = 0–0.51%; Mann–Whitney *p* = 0.0010) (Figure 3D). *Campylobacter* (ASV 161) had a significant moderate negative correlation with fecal UDCA concentration (Spearman *ρ* = −0.3806, *p* = 0.0264) (Figure 3E). *Campylobacter* (ASV 161) was not detected in any of the healthy cats but was detected in 13/28 of cats with CKD (median relative abundance = 0%; range = 0–0.62%; Mann–Whitney *p* = 0.0663) (Figure 3F).

### 3.3 A subpopulation of CKD cats experience fecal bile acid dysmetabolism with <50% secondary bile acids

**Figure 4:**
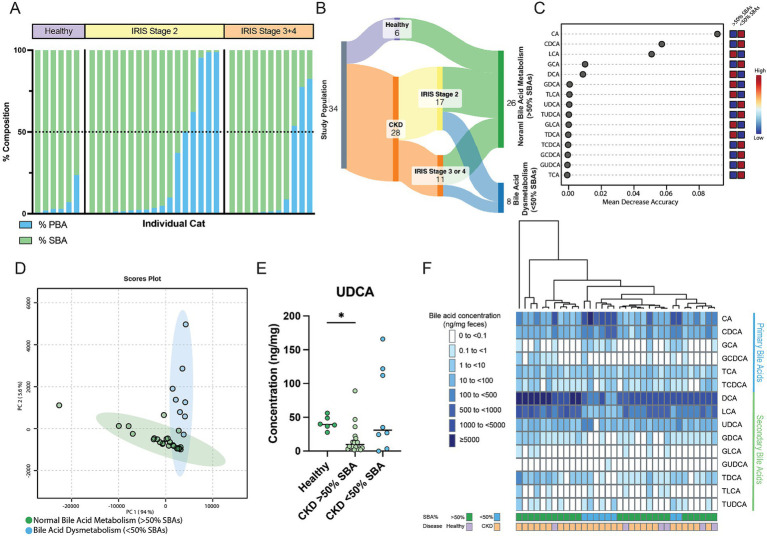
Distinct fecal bile acid pool composition in CKD cats with bile acid dysmetabolism characterized by <50% SBAs. **(A)** Stacked bar chart demonstrating the composition of PBAs (blue) and SBAs (green) in the feline fecal bile acid pool. A horizontal dotted line demarcates 50% composition. Each bar along the x-axis represents an individual cat, with individuals grouped by healthy or IRIS CKD Stage. **(B)** Sankey diagram demonstrating cats within normal bile acid metabolism (>50% SBAs) and bile acid dysmetabolism (<50% SBAs) groups. **(C)** Random Forest machine learning algorithm with dots corresponding to a fecal bile acid along the left-hand y-axis and the listed mean decrease in accuracy along the x-axis. Boxes along the right-hand y-axis demonstrate whether the fecal bile acid is high (red) or low (blue) in normal bile acid metabolism (>50% SBAs) and bile acid dysmetabolism (<50% SBAs) groups. **(D)** Principal component analysis of fecal bile acid concentrations in CKD cats with normal bile acid metabolism (>50% SBAs) (individual samples represented by green points) and CKD cats with bile acid dysmetabolism (<50% SBAs) (individual samples represented by blue points) and a 95% confidence interval clouds for the clustering of each group. Variance explained by each principal component is listed on the corresponding axis. **(E)** Fecal concentration of UDCA in healthy cats, CKD cats with normal bile acid metabolism (>50% SBAs), and CKD cats with bile acid dysmetabolism (<50% SBAs); significance determined via Kruskal-Wallis with FDR adjustment, significant discoveries noted by *. **(F)** Unsupervised hierarchical clustering of fecal bile acid composition. Individual samples are in columns and are colored along the bottom x-axis by both percentage of SBA and disease status. Each row represents a single bile acid listed along the right-hand y-axis. Shade of blue within a square corresponds to the concentration (ng/mg of feces) of each bile acid, with breakpoint concentrations by color shown at the left-hand side of the figure mat. Dendrogram at the top represents relative sample similarity determined by hclust in R Studio.

Within cats with CKD, 29% (8/28) had a fecal bile acid dysmetabolism characterized by the total bile acid composition of <50% SBAs detected (Figure 4A). By contrast, no healthy cats had a composition of fecal bile acids with <50% SBAs. To further investigate this difference, the subpopulation of CKD cats with fecal bile acid dysmetabolism (<50% SBAs) (*n* = 8) were compared to healthy and CKD cats with normal bile acid metabolism (>50% SBAs) (*n* = 26) (Figure 4B). Using Random Forest machine learning algorithm, fecal concentrations of the host derived PBAs CA and CDCA were best able to discriminate between cats with normal bile acid metabolism (defined as >50% SBAs) and those with a bile acid dysmetabolism (<50% SBAs) (Figure 4C). Based on principal component analysis, distinct clustering of the cats with normal bile acid metabolism (>50% SBAs) and those CKD cats with bile acid dysmetabolism (<50% SBAs) is demonstrated (Figure 4D). The relationship of reduced fecal concentration of UDCA in CKD cats was reexamined by comparing healthy cats to CKD cats with normal bile acid metabolism (>50% SBAs) and CKD cats with bile acid dysmetabolism (<50% SBAs). The fecal UDCA concentrations remained significantly reduced in the population of CKD cats with normal bile acid metabolism (>50% SBAs) (median 9.8 ng/mg; FDR adj. *p* = 0.0092) compared to healthy cats (median 39.4 ng/mg) (Figure 4E). However, the fecal concentration of UDCA in CKD cats with bile acid dysmetabolism (<50% SBAs) was not significantly different from healthy cats (median 31.05 ng/mg, FDR adj. *p* = 0.2303) (Figure 4E). In total, using unsupervised hierarchical clustering, the total composition of fecal bile acids clustered by SBA percentage as opposed to disease status (Figure 4F).

### 3.4 CKD cats with bile acid dysmetabolism (<50% SBAs) have a distinct gut microbial community structure

**Figure 5:**
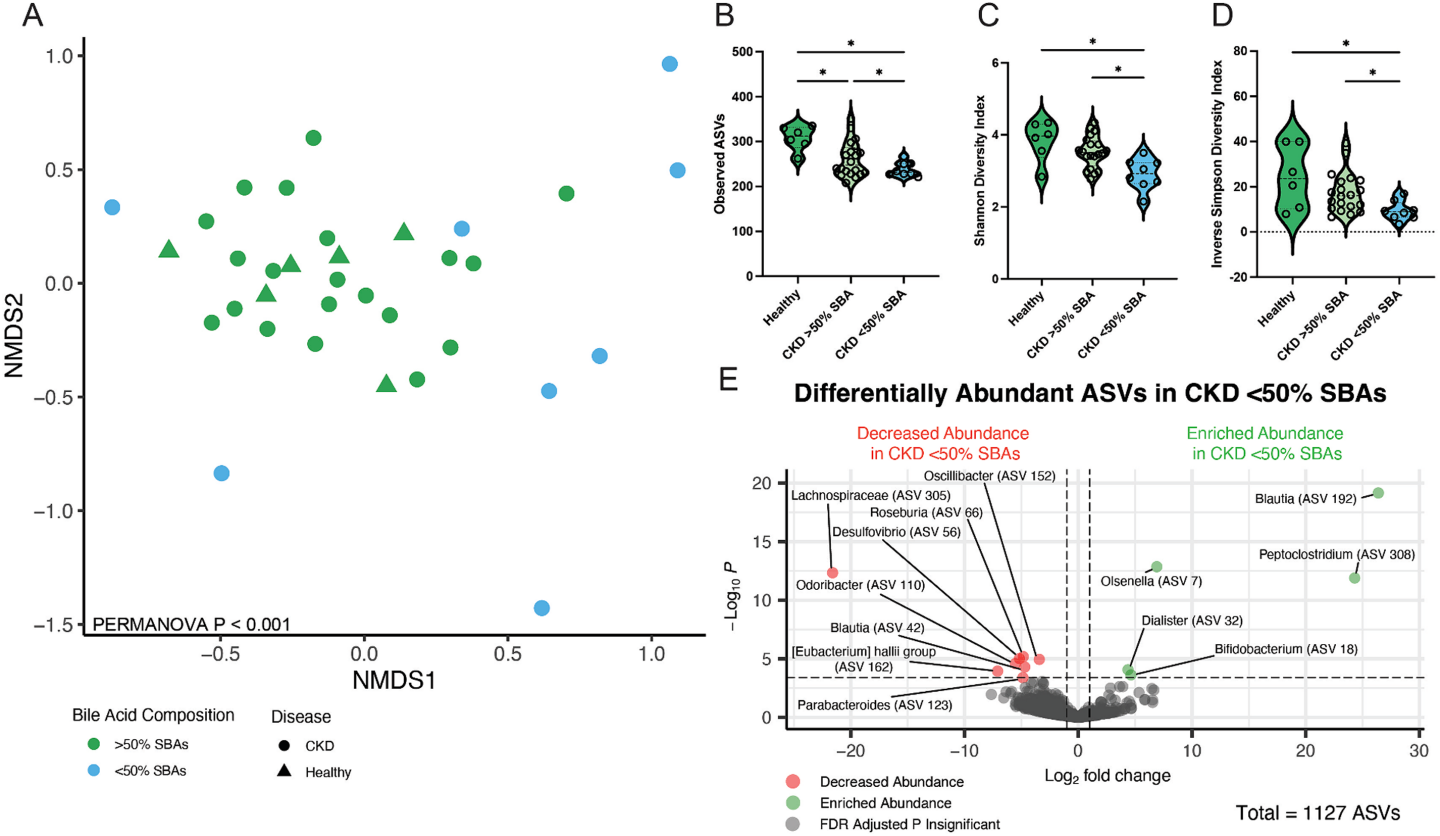
Fecal microbial population from CKD cats with bile acid dysmetabolism (<50% SBAs) is distinct from healthy cats and CKD cats with normal bile acid metabolism (>50% SBAs). **(A)** Beta diversity determined with Bray–Curtis dissimilarity distances depicted with non-metric multi-dimensional scaling (NMDS) plot with individual samples represented by points for cats with normal bile acid metabolism (>50% SBAs; green) and bile acid dysmetabolism (<50% SBAs; blue) and significance determined with PERMANOVA. **(B)** Alpha diversity in healthy cats, CKD cats with normal bile acid metabolism (>50% SBAs), and CKD cats with bile acid dysmetabolism (<50% SBAs) represented by violin plots of total observed ASVs and significance determined via parametric one-way ANOVA with FDR adjustment; significant discoveries noted by *. **(C)** Alpha diversity in healthy cats, CKD cats with normal bile acid metabolism (>50% SBAs), and CKD cats with bile acid dysmetabolism (<50% SBAs) represented by violin plots of Shannon Diversity Index and significance determined via parametric one-way ANOVA with FDR adjustment; significant discoveries noted by *. **(D)** Alpha diversity in healthy cats, CKD cats with normal bile acid metabolism (>50% SBAs), and CKD cats with bile acid dysmetabolism (<50% SBAs) represented by violin plots of Inverse Simpson Diversity Index and significance determined via parametric one-way ANOVA with FDR adjustment; significant discoveries noted by *. **(E)** Volcano plot displaying differentially abundant ASVs determined with DESeq2 that are either reduced in abundance (red) or enriched in abundance (green) in CKD cats with bile acid dysmetabolism (<50% SBAs) compared to healthy cats and CKD cats with normal bile acid metabolism (>50% SBAs). Points maintaining significance following FDR adjustment (*p* < 0.05) and having a log2FC of at least magnitude 2 are plotted and colored. All gray points do not meet these criteria.

The microbial community structure of CKD cats with bile acid dysmetabolism (<50% SBAs) was compared to cats with normal bile acid metabolism (>50% SBAs) including healthy and a subset of CKD cats (Figure 5A). Alpha diversity assessed by total ASVs, Shannon Diversity Index, and Inverse Simpson Diversity Index were all significantly reduced in cats with CKD with bile acid dysmetabolism (<50% SBAs) when compared to both healthy cats (FDR adj. *p* = 0.006, 0.009, and 0.0096, respectively) and CKD cats with normal bile acid metabolism (>50% SBAs) (FDR adj. *p* = 0.0427, 0.0017, and 0.0438, respectively) (Figures 5B–D). Beta diversity assessed with Bray–Curtis dissimilarity distances was significantly different in CKD cats with bile acid dysmetabolism (<50% SBAs) when compared to both healthy cats and CKD cats with normal bile acid metabolism (>50% SBAs) (PERMANOVA *p* < 0.001) (Figure 5A). Differentially abundant ASVs were determined with DESeq2 when CKD cats with bile acid dysmetabolism (<50% SBAs) were compared to healthy and CKD cats with normal bile acid metabolism (>50% SBAs). *Lachnospiraceae* (ASV 305) was significantly reduced in CKD cats with bile acid dysmetabolism (<50% SBAs) (log2FC = −21.6, FDR adj. *p* < 0.0001). Sorted by FDR adjusted *p*-value, the next three ASVs also found to be significantly reduced were *Roseburia* sp. (ASV 66) (log2FC = −4.8, FDR adj. *p* = 0.0013), *Oscillibacter* sp. (ASV 152) (log2FC = −3.4, FDR adj. *p* = 0.0016), and *Desulfovibrio* sp. (ASV 56) (log2FC = −5.2, FDR adj. p = 0.0016) (Figure 5E). The top three ASVs enriched in CKD cats with bile acid dysmetabolism (<50% SBAs) were *Blautia* sp. (ASV 192) (log2FC = 26.4, FDR adj. *p* < 0.0001), *Olsenella* sp. (ASV 7) (log2FC = 6.9, FDR adj. p < 0.0001), and *Peptoclostridium* sp. (ASV 308) (log2FC = 24.3, FDR adj. *p* < 0.0001) (Figure 5E). The *Peptoclostridium* sp. (ASV 308) was also investigated for sequence similarity to *P. hiranonis* with NCBI Blast and found to have 99.58% sequence identity (Table 1).

**Figure 6:**
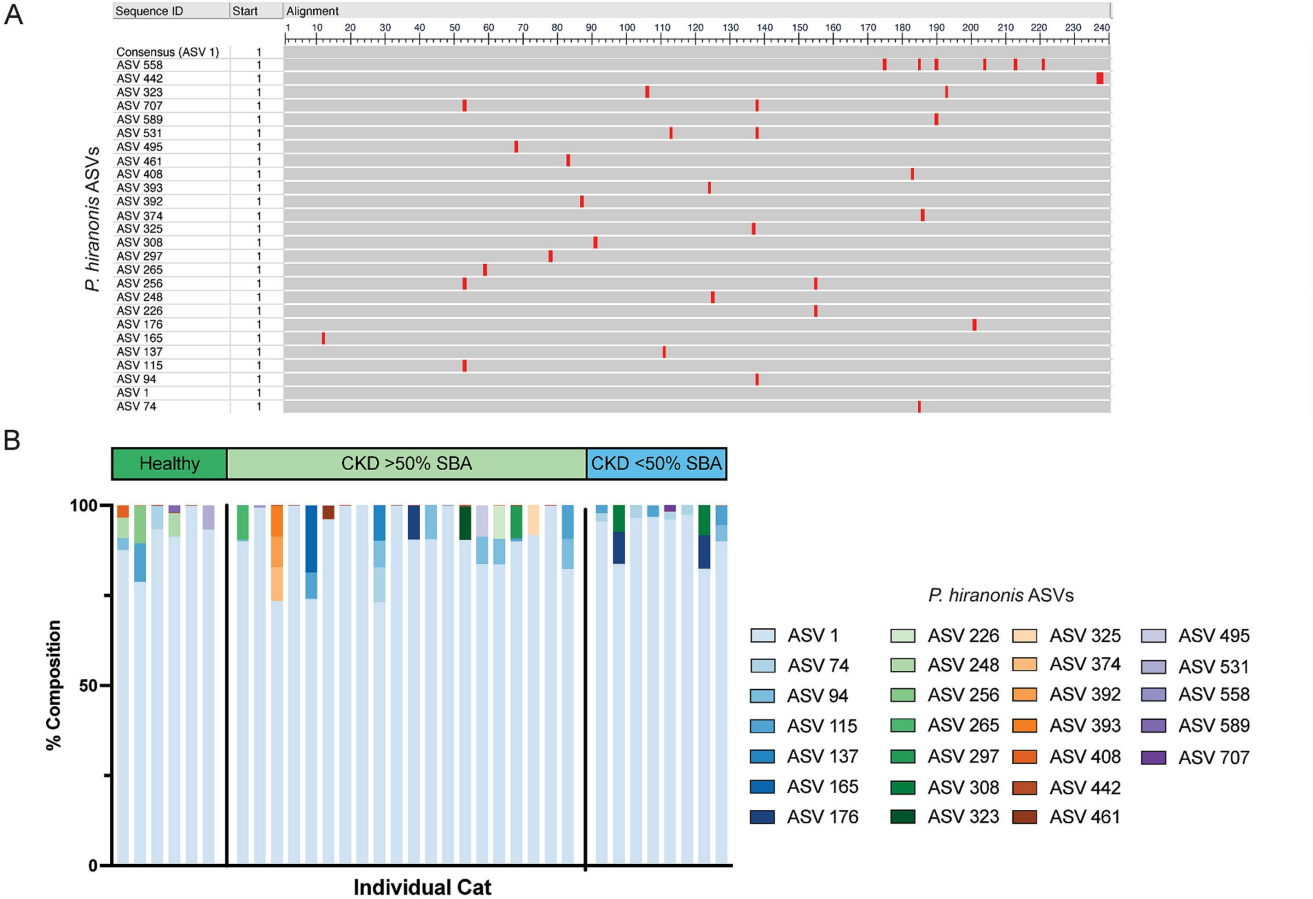
26 ASVs with at least 97% nucleotide identity to *P. hiranonis* are identified in the fecal microbiota of cats. **(A)** Multiple sequence alignment of the 26 ASVs with at least 97% sequence identity to *P. hiranonis*. ASV 1 was used as the consensus sequence. Red bars indicate the location in the 240 base pair sequence of amplicons that nucleotide variation from the consensus sequence occurs within the V4 region of the 16S rRNA gene. **(B)** Stacked bar plot representing the relative composition of all 26 ASVs identified with at least 97% sequence identity to *P. hiranonis* in individual cat fecal samples.

Given that *P. hiranonis* produces SBAs via the *bai* operon, it was unexpected for an ASV with sequence similarity to this organism to have increased relative abundance in cats with reduced SBAs; thus, all ASVs with a family level taxonomic assignment of *Peptostreptococcaceae* (*n* = 65 ASVs) were screened with NCBI Blast for sequence similarity to *P. hiranonis* (Table 1). A total of 26 ASVs with at least 97% sequence identity to the *P. hiranonis* complete genome isolate in NCBI were identified. Multiple sequence alignment using ASV 1 (identified as with *P. hiranonis*) as the consensus sequence was performed to visualize locations of nucleotide variation with sequences from the V4 region of the 16S rRNA gene amongst the 26 ASVs consistent with *P. hiranonis* (Figure 6A). Additionally, the proportion of which ASVs made up the composition of total ASVs with at least 97% sequence identity to *P. hiranonis* within a single fecal sample were also visualized, with ASV 1 contributing at minimum 73% of all *P. hiranonis* relative abundance to all fecal samples (Figure 6B).

### 3.5 Reduced relative abundance of *Peptacetobacter hiranonis*, *Roseburia*, and *Oscillospirales* ASVs in CKD cats with bile acid dysmetabolism (<50% SBAs) correlate with fecal bile acid composition

**Figure 7:**
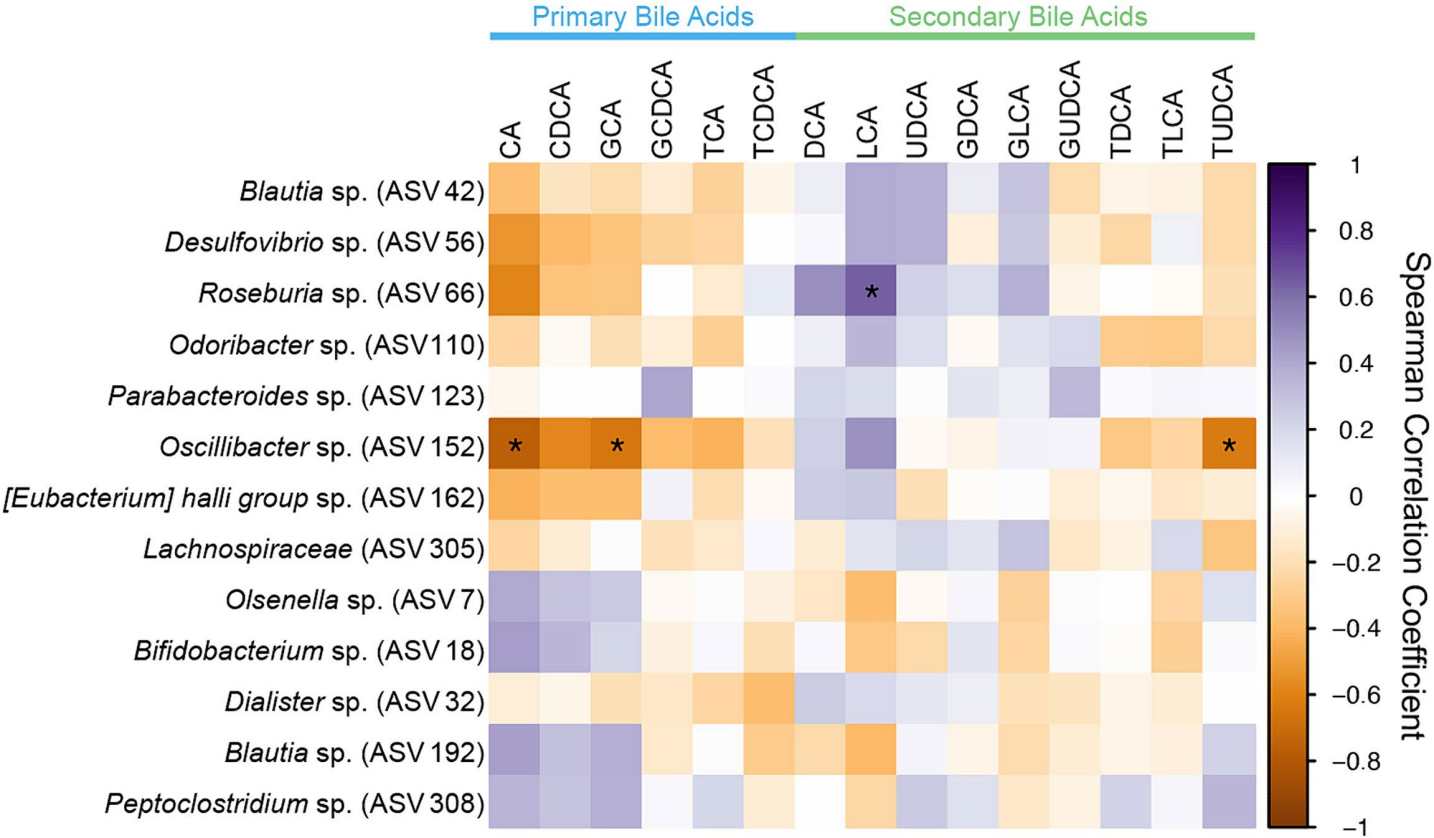
Spearman correlation matrix of fecal bile acid concentrations for individual bile acids and differentially abundant ASVs identified in CKD cats with bile acid dysmetabolism (<50% SBAs) using DESeq2. Spearman *ρ* for each pair of fecal bile acid concentration and each ASV relative abundance denoted by color of the square at the corresponding intersection. Squares denoting positive correlations are purple, with darker shades indicating stronger positive correlations. Squares denoting negative correlations are orange, with darker shades indicating stronger negative correlations. * denotes FDR adjusted *p* < 0.05.

All ASVs identified as differentially abundant in CKD cats with bile acid dysmetabolism (<50% SBAs) were assessed for significant correlations with the fecal bile acid composition (Figure 7). Of those, *Roseburia* (ASV 66) was significantly positively correlated with the SBA LCA (Spearman *ρ* = 0.6442, FDR adj. *p* = 0.0150). *Roseburia* sp. (ASV 66) amplicon sequence had 98.75% sequence identity to *Roseburia intestinalis* when compared to the reference 16S rRNA gene sequence with NCBI Blast. Additionally, *Oscillibacter* sp. (ASV 152) was significantly negatively correlated with the PBAs CA (Spearman ρ = −0.7551, FDR adj. *p* = 0.0003) and GCA (Spearman *ρ* = −0.6443, FDR adj. *p* = 0.0150) and the SBA TUDCA (Spearman *ρ* = −0.6229, FDR adj. *p* = 0.0238).

Given that *Oscillibacter* taxonomically is within the order *Oscillospirales* and family *Oscillospiraceae*, which have metagenomically been described to contain the *bai* operon and possibly contribute to SBA production (Vital et al., 2019; Kim et al., 2022), the dataset was further screened for taxonomically similar organisms with similar correlation pattern to the fecal bile acid composition as *Oscillibacter.* This screening produced two additional ASVs within the order *Oscillospirales* (Table 2), one uncultured *UBA1819* genus within the *Ruminococcaceae* family (ASV 157) and one *Colidextribacter* sp. (ASV 186).

**Table 2:** ASVs from the order *Oscillospirales* with significant correlations to fecal bile acid composition in cats.

ASV ID	Range % Relative Abundance	V4 region of 16S rRNA Gene Amplicon Sequence 5′-3’	Silva Taxonomy (v138.1)	NCBI BLAST Result(s)	Nucleotide Identity (%)
152	0–0.34%	TACGTAGGTGGCAAGCGTTGTCCGGATTTACTGGGTGTAAAGGGCGTGCAGCCGGGCCGGCAAGTCAGATGTGAAATCTGGAGGCTTAACCTCCAAACTGCATTTGAAACTGTAGGTCTTGAGTACCGGAGAGGTTATCGGAATTCCTTGTGTAGCGGTGAAATGCGTAGATATAAGGAAGAACACCAGTGGCGAAGGCGGATAACTGGACGGCAACTGACGGTGAGGCGCGAAAGCGTG	Order: Oscillospirales Family: Oscillospiraceae Genus: *Oscillibacter*	*Oscillibacter* sp. PEA192 DNA, complete genome Dysosmobacter welbionis strain J115 chromosome, complete genome	100% 100%
157	0–1.26%	AACGTAGGGTGCAAGCGTTGTCCGGAATTACTGGGTGTAAAGGGAGCGCAGGCGGATTGGCAAGTTGGGAGTGAAATCTATGGGCTCAACCCATAAATTGCTTTCAAAACTGTCAGTCTTGAGTGGTGTAGAGGTAGGCGGAATTCCCGGTGTAGCGGTGGAATGCGTAGATATCGGGAGGAACACCAGTGGCGAAGGCGGCCTACTGGGCACTAACTGACGCTGAGGCTCGAAAGCATG	Order: Oscillospirales Family: Ruminococcaceae Genus: UBA1819	*Ruthenibacterium lactatiformans* isolate	100%
186	0–0.33%	TACGTAGGTGGCAAGCGTTATCCGGATTTACTGGGTGTAAAGGGCGTGTAGGCGGGATCGCAAGTCAGATGTGAAAACTGGAGGCTCAACCTCCAGCCTGCATTTGAAACTGTGGTTCTTGAGTACTGGAGAGGCAGACGGAATTCCTAGTGTAGCGGTGAAATGCGTAGATATTAGGAGGAACACCAGTGGCGAAGGCGGTCTGCTGGACAGCAACTGACGCTGAGGCGCGAAAGCGTG	Order: Oscillospirales Family: Ruminococcaceae Genus: *Colidextribacter*	*Clostridiales bacterium* CCNA10 DNA, complete genome *Flintibacter butyricus* partial 16S rRNA gene	100% 100%

**Figure 8:**
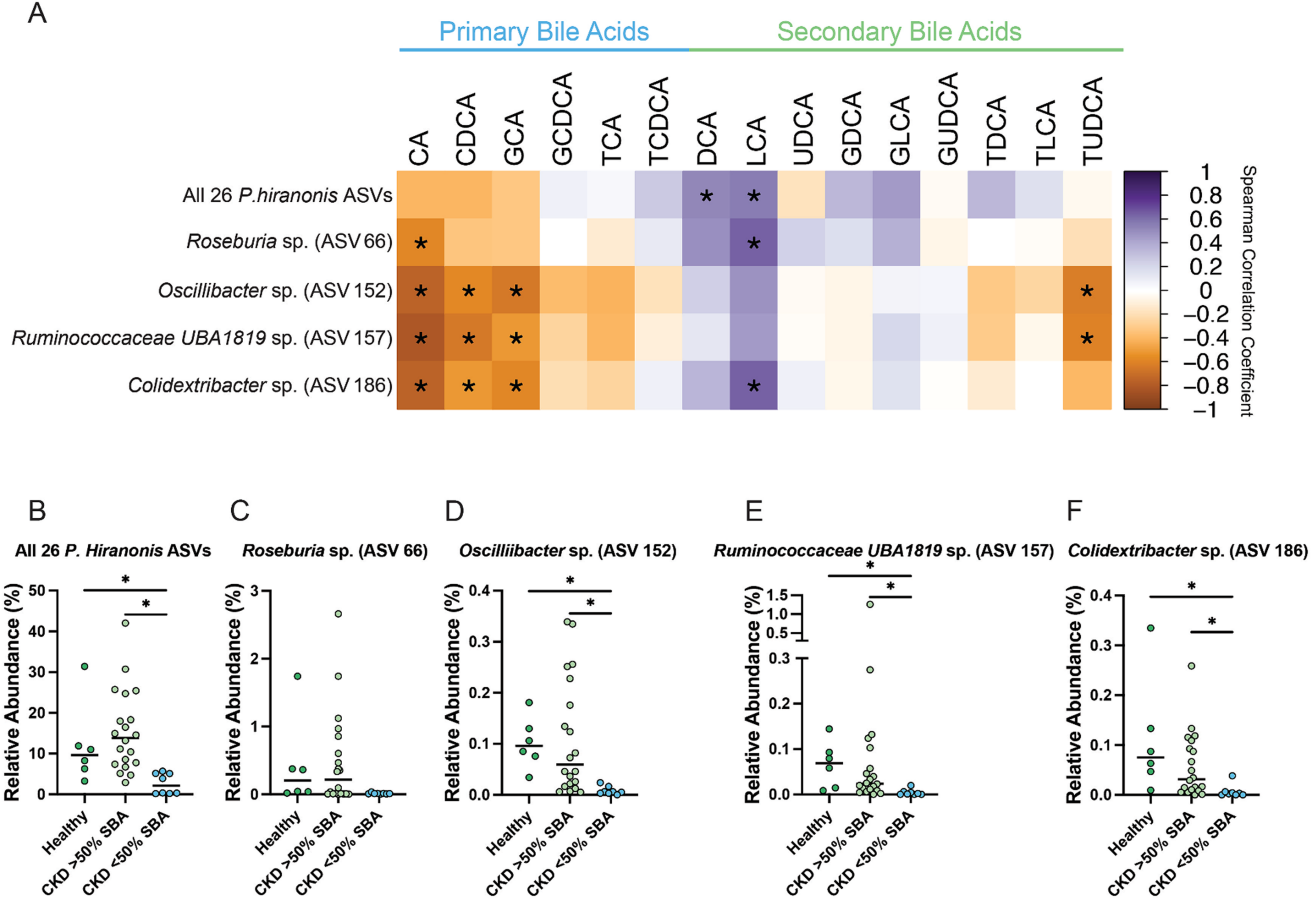
Fecal bile acid composition correlates with differentially abundant *P. hiranonis*, *Roseburia* sp., and *Oscillospirales* ASVs. **(A)** Spearman correlation matrix of fecal bile acid concentrations for individual bile acids with the relative abundance of 26 combined *P. hiranonis* ASVs, *Roseburia* sp. (ASV 66), *Oscillibacter* sp. (ASV 152), *Ruminococcaceae UBA1819* (ASV 157), and *Colidextribacter* sp. (ASV 186). Spearman *ρ* for each pair of fecal bile acid concentration and ASV relative abundance denoted by color of the square at the corresponding intersection. Squares denoting positive correlations are purple, with darker shades indicating stronger positive correlations. Squares denoting negative correlations are orange, with darker shades indicating stronger negative correlations. * denotes FDR adjusted *p* < 0.05. Relative abundance in healthy cats, CKD cats with normal bile acid metabolism (>50% SBAs), and CKD cats with bile acid dysmetabolism (<50% SBAs) for **(B)** All 26 *P. hiranonis* ASVs, **(C)** *Roseburia* sp. (ASV 66), **(D)** *Oscillibacter* sp. (ASV 152), **(E)** *Ruminococcaceae UBA1819* (ASV 157), and **(F)** *Colidextribacter* sp. (ASV 186). Significance determined via Kruskal-Wallis with FDR adjustment; significant discoveries noted by *.

These two ASVs, along with the previously identified *Oscillibacter* sp. (ASV 152) and *Roseburia* sp. (ASV 66), as well as the total relative abundance of the 26 ASVs with at least 97% sequence identity to *P. hiranonis* were all assessed for correlation with the fecal bile acid composition (Figure 8A). The total relative abundance of the 26 *P. hiranonis* ASVs had significant positive correlation with the SBAs DCA (Spearman *ρ* = 0.5218, FDR adj. *p* = 0.0407) and LCA (Spearman ρ = 0.5615, FDR adj. *p* = 0.0156). The relative abundance of *P. hiranonis* ASVs was significantly reduced in CKD cats with bile acid dysmetabolism (<50% SBAs) (median relative abundance = 2.1%; range = 0.21–5.67%) compared to CKD cats with normal bile acid metabolism (>50% SBAs) (median relative abundance = 13.9%; range = 2.92–42.02%; FDR adj. *p* = 0.0002) and healthy cats (median relative abundance = 9.7%; range = 3.27–31.38%; FDR adj. *p* = 0.0112) (Figure 8B).

*Roseburia* sp. (ASV 66) relative abundance had a significant negative correlation with the PBA CA (Spearman *ρ* = −0.5841, FDR adj. *p* = 0.0105) and significant positive correlation with the SBA LCA (Spearman *ρ* = 0.6442, FDR adj. *p* = 0.0023) (Figure 8A). The relative abundance of *Roseburia* sp. (ASV 66) in CKD cats with bile acid dysmetabolism (<50% SBAs) (median relative abundance = 0.011%; range = 0.0031–0.036%) was not statistically significantly reduced compared to CKD cats with normal bile acid metabolism (>50% SBA) (median relative abundance = 0.22%; range = 0–2.66%; FDR adj. *p* = 0.0674) or healthy cats (median relative abundance = 0.20%; range = 0.019–1.72%; FDR adj. *p* = 0.0600) (Figure 8C).

*Oscillibacter* sp. (ASV 152) relative abundance had a significant negative correlation with the PBAs CA (Spearman *ρ* = −0.7551, FDR adj. *p* < 0.0001), CDCA (Spearman *ρ* = −0.5719, FDR adj. *p* = 0.0126), and GCA (Spearman *ρ* = −0.6443, FDR adj. *p* = 0.0023) as well as the SBA TUDCA (Spearman *ρ* = −0.6229, FDR adj. *p* = 0.0038) (Figure 8A). The relative abundance of *Oscillibacter* sp. (ASV 152) was significantly reduced in CKD cats with bile acid dysmetabolism (<50% SBAs) (median relative abundance = 0.0057%; range = 0–0.024%) compared to CKD cats with normal bile acid metabolism (>50% SBAs) (median relative abundance = 0.059%; range = 0.0052–0.34%; FDR adj. *p* = 0.0008) and healthy cats (median relative abundance = 0.096%; range = 0.034–0.18%; FDR adj. *p* = 0.0009) (Figure 8D).

*Ruminococcaceae UBA1819* sp. (ASV 157) relative abundance had a significant negative correlation with the PBAs CA (Spearman *ρ* = −0.8176, FDR adj. *p* < 0.0001), CDCA (Spearman *ρ* = −0.6475, FDR adj. *p* = 0.0023), and GCA (Spearman ρ = −0.5143, FDR adj. *p* = 0.0428) as well as the SBA TUDCA (Spearman *ρ* = −0.6071, FDR adj. *p* = 0.0058) (Figure 8A). The relative abundance of *Ruminococcaceae UBA1819* sp. (ASV 157) was significantly reduced in CKD cats with bile acid dysmetabolism (<50% SBAs) (median relative abundance = 0.0016%; range = 0–0.20%) compared to CKD cats with normal bile acid metabolism (>50% SBAs) (median relative abundance = 0.024%; range = 0–1.26%; FDR adj. *p* = 0.0011) and healthy cats (median relative abundance = 0.069%; range = 0.0083–0.14%; FDR adj. *p* = 0.0011) (Figure 8E).

*Colidextribacter* sp. (ASV 186) relative abundance had a significant negative correlation with the PBAs CA (Spearman *ρ* = −0.7560, FDR adj. *p* < 0.0001), CDCA (Spearman ρ = −0.5188, FDR adj. *p* = 0.0409), and GCA (Spearman *ρ* = −0.5809, FDR adj. *p* = 0.0105) as well as a significant positive correlation with the SBA LCA (Spearman *ρ* = 0.6417, FDR adj. *p* = 0.0023) (Figure 8A). The relative abundance of *Colidextribacter* sp. (ASV 186) was significantly reduced in CKD cats with bile acid dysmetabolism (<50% SBAs) (median relative abundance = 0.0026%; range = 0–0.038%; range = 0–0.26%) compared to CKD cats with normal bile acid metabolism (>50% SBAs) (median relative abundance = 0.0052%, FDR adj. *p* = 0.0022) and healthy cats (median relative abundance = 0.075%; range = 0.0094–0.33%; FDR adj. *p* = 0.0014) (Figure 8F).

## 4 Discussion

Here, it is reported that cats with CKD have an altered fecal bile acid profile compared to apparently clinically healthy cats. The altered fecal bile acid profile is characterized by a reduced concentration of the microbial-derived SBA UDCA. Even when a subpopulation of CKD cats with bile acid dysmetabolism (<50% SBAs) was separated, the presence of reduced fecal UDCA concentrations in CKD cats with normal bile acid metabolism (>50% SBAs) compared to healthy cats remained. The reduction in UDCA is associated with differentially abundant microbes, notably reduced relative abundance of a *Lachnospiraceae* sp. (ASV 286) and an uncultured *Clostridia* sp. (ASV 151). Other members within these taxonomic classifications are known to perform both the deconjugation function via BSH (Foley et al., 2019; Lucas et al., 2021) as well as utilize HSDH enzymes to generate SBAs from PBAs (Lucas et al., 2021; Sutherland and Williams, 1985; Coleman et al., 1994; Lou et al., 2016). Both of those biotransformations are crucial for the microbial production of the SBA UDCA. While not possible with the 16S rRNA gene amplicon sequencing performed in this study, future analysis employing metagenomics could profile genetic capacities to provide mechanistic insight into functional potential of the feline gut microbiome in CKD cats and potentially causative support for the correlative findings described herein. Additionally, it is also described here that the enteropathogen *Campylobacter* is detected within feces of cats with CKD and its increasing relative abundance negatively correlates with fecal UDCA concentration. The clinical significance of these findings are unknown and require further investigation, though it is noted that *Campylobacter* is also described as enriched in abundance from a metagenomic characterization of the gut microbiota of obese cats (Ma et al., 2022).

Reduced fecal concentrations of UDCA in cats with CKD is notable given the signaling activity profile of UDCA to host cells. UDCA can be directly sensed by the host as agonist for the cell surface expressed bile acid activated receptor TGR5 as well as an antagonist of the nuclear receptor FXR (Ticho et al., 2019). While expression is yet to be described in cats, both TGR5 and FXR are known to be expressed in the kidneys of humans and rodents (Herman-Edelstein et al., 2018). UDCA can act to modulate and mitigate inflammation in both acute and chronic settings through multiple mechanisms (Poupon, 2012). This had led to exploring its adjunctive use as an anti-inflammatory to treat human patients with SARS-CoV-2 infection (Brevini et al., 2023) and colitis (Ward et al., 2017) in addition to its original purpose as a therapy for primary biliary cirrhosis (Goulis et al., 1999). The anti-fibrotic properties of UDCA have recently been linked to inhibition of cellular autophagy *in vitro* in a liver fibrosis model (Ye et al., 2020). Given the chronic inflammatory and fibrosing pathophysiology of CKD, it is important to determine if reduced microbial production of UDCA can exacerbate CKD. It has been reported that other TGR5 agonists both inhibit development of kidney disease in murine models of obesity and diabetes (Wang et al., 2016), and more recently that a TGR5 agonist produced by the gut microbe *Bacteroides fragilis* can ameliorate renal fibrosis in both unilateral ureteral obstruction and adenine-induced CKD murine models (Zhou et al., 2022). In a mouse model of cisplatin induced acute kidney injury, UDCA protects the murine kidney from developing kidney injury by limiting oxidative damage and preserving mitochondrial function (Yang et al., 2020). UDCA is similarly protective in a gentamicin-induced rodent nephrotoxicity model by modulating NF-κB mediated inflammation (Abd-Elhamid et al., 2018). UDCA is commercially available as Ursodiol and is a widely utilized therapeutic in both human and veterinary medicine (Poupon et al., 1991; Otte et al., 2013), making UDCA a potentially beneficial adjunctive therapy for CKD treatment from a cytoprotective, anti-fibrotic, and anti-inflammatory perspective. The data presented here suggest that cats with CKD may represent a spontaneous translational disease model where the therapeutic potential of modulation of bile acid metabolism can be further explored. Importantly, to determine whether a reduced fecal concentration of UDCA in cats with CKD is biologically relevant in CKD pathophysiology, additional information characterizing circulating serum concentrations of UDCA, uptake of UDCA by the host kidney, and UDCA’s ability to interact with bile acid activated receptors in the kidney is required.

It is also reported here that a subpopulation of cats with CKD experience a fecal bile acid dysmetabolism characterized by fecal bile acid profile with <50% SBAs. In people (Ridlon et al., 2006) and in dogs (Rowe and Winston, 2024), the fecal bile acid pool is typically composed of >80–90% microbial-derived SBAs in states of health. Fecal bile acid profiles have been minimally characterized in cats (Rowe and Winston, 2024), with three studies performed in the context of dietary impact on fecal bile acid profiles (Anantharaman-Barr et al., 1994; Jackson et al., 2020; Ephraim and Jewell, 2021), three studies in the context of antimicrobial impact (Whittemore et al., 2018; Whittemore et al., 2019; Stavroulaki et al., 2022), and a single study investigating CKD and dietary impact (Hall et al., 2020). From these studies, the primary association leading to a bile acid dysmetabolism and decreased microbial-derived SBAs is the administration of antimicrobials, which causes reduced microbiota diversity and can persist for at least 6 weeks based on one study of cats administered clindamycin (Whittemore et al., 2019). In the present study, cats were excluded if they received antimicrobials within 6 weeks of enrollment. However, it is possible that antimicrobial administration prior to 6 weeks before enrollment could create a persistent dysbiosis longer than 6 weeks. Current longitudinal data of healthy research cats demonstrates persistent changes in microbial diversity following clindamycin can persist for 630 days, even though the SBA DCA normalizes by that same time (Whittemore et al., 2018). Diet is also a major driver of gut microbiota composition (Pilla and Suchodolski, 2021), so it is possible that lack of dietary controls in the present study is contributing to the heterogeneity of fecal bile acid profiles seen in both apparently clinically healthy cats and cats with CKD. Moreover, it is recently reported that cats with underlying gastrointestinal disease can have fecal bile acid dysmetabolism characterized by <50% SBAs (Sung et al., 2023). So, while effort was made to exclude cats in the present study with primary gastrointestinal disease, it is possible that unidentified primary gastrointestinal disease could also have been a contributing factor, especially with the known difficulty of clinical signs alone to exclude the presence of underlying gastrointestinal pathology in cats (Marsilio et al., 2019). Ultimately, it is not possible from the data presented here to determine a singular driver leading to the subpopulation of CKD cats with bile acid dysmetabolism (<50% SBAs) but this could indicate distinct populations of CKD cats.

Still, it is important to explore the gut microbial community structure alterations corresponding with the subpopulation of CKD cats with bile acid dysmetabolism (<50% SBAs) as they may represent a clinically important and relevant disease state. The largest driver for conversion of host PBAs into microbial-derived SBAs is the 7α-dehydroxylation (Funabashi et al., 2020). This process conferred through the *bai* operon is known to be present in only a select few culturable microbes: *Clostridium scindens* (Kitahara et al., 2000), *Clostridium hylemonae* (Ridlon et al., 2010), *Peptacetobacter hiranonis* (formerly *Clostrdium hiranonis*) (Kitahara et al., 2001), and *Extibacter muris* (in mice) (Streidl et al., 2021). Recently, *Roseburia intestinalis* has been suggested to contain only a portion of the *bai* operon and provide a minor contribution of 7α-dehydroxylation activity when cultured *in vitro* with CA (Lucas et al., 2021). From the present study, a single *Roseburia* sp. (ASV 66) with 98.75% sequence identity to *Roseburia intestinalis* to the V4 region of 16S rRNA gene negatively correlated with CA and positively correlated with the SBA LCA. Of known full *bai* operon-containing organisms, only *P. hiranonis* has been identified in cats, and is currently used in a validated assay to assess dysbiosis in cats with chronic enteropathy (Sung et al., 2022). Here, 26 ASVs were identified as having 97% nucleotide identity or greater with the V4 region of 16S rRNA gene of *P. hiranonis* when assessed against the full genome in the NCBI database. Of those ASVs, some were identified in relative abundances as high as 30%, while others were found in relative abundances of less than 1% (Figure 6B). When all 26 *P. hiranonis* ASVs are combined and assessed for correlation with fecal bile acid composition, the total *P. hiranonis* ASVs were significantly positively correlated with the concentrations of SBAs DCA and LCA. There was also significantly reduced relative abundance of these *P. hiranonis* ASVs in CKD cats with bile acid dysmetabolism (<50% SBAs). Taken together, it appears that *P. hiranonis* is likely an important producer of SBAs in cats, and this function can be lost in states of dysbiosis leading to fecal bile acid dysmetabolism.

Here it is also identified that three ASVs belonging to the order *Oscillospirales* also either significantly negatively correlated with PBA concentration or are significantly positively correlated with SBA concentration. This correlation pattern is similar to the pattern demonstrated by the known 7α-dehydroxylation performing *P. hiranonis*. The relative abundances of all three *Oscillospirales* ASVs were significantly reduced in CKD cats with a fecal bile acid dysmetabolism characterized by a PBA predominant fecal bile acid profile. Recently, human gut metagenome assembled genome (MAG) data, describes unculturable members of *Oscillospirales* that harbor the *bai* operon and contribute to 7α-dehydroxylation (Vital et al., 2019; Kim et al., 2022). These microbes also contain the genetic potential to generate secondary *allo*-bile acids via a direct pathway without the involvement of other microbial transformations (Lee et al., 2022; Ridlon et al., 2023). These recent findings highlight members of the family *Oscillospirales* as novel microbes to perform bile acid biotransformation within the human gut microbiota. Further, in human inflammatory bowel disease patients two ASVs identified via 16S rRNA gene amplicon sequencing as uncultured *Oscillospiraceae* positively correlated in relative abundance with fecal concentrations of the SBAs DCA and LCA (Lavelle et al., 2022). The presence and/or function of *Oscillospirales* as possible novel bile acid converting organisms in other species besides humans is currently not described in the literature. The data presented here are not causative but suggest similar or the same microbes identified by metagenomics of human gut microbes are also present within the gut microbiota of cats and thus may contribute to PBA conversion into SBAs in some capacity. Further investigation of bile acid dysmetabolism in cats via paired metagenomic sequencing and targeted metabolomics will allow for interrogation of the correlative data presented here that suggest a relationship may exist between *Oscillospirales* and microbial bile acid metabolism in cats.

Notably, when fecal UDCA concentrations are considered in CKD cats with <50% SBAs, a heterogeneity of concentrations is observed. UDCA production is mechanistically not tied to 7α-dehydroxylation directly, but rather can be achieved through a variety of reactions including hydroxysteroid dehydrogenase (HSDH) enzymes that act at the 7^th^ carbon position. This enzymatic activity is provided by many members of the gut microbiota (Rowe and Winston, 2024). It is possible that a heterogenous spread of fecal UDCA concentrations are present in CKD cats with <50% SBAs given the greater availability of PBAs to undergo modification via non 7α-dehydroxylation methods, including through HSDH enzymes. Further exploration of this phenomenon may be better explained by incorporation of metagenomic sequencing to identify gut microbiota members with bile acid biotransformation genes and thorough evaluation of the fecal bile acid pool beyond the 15 BAs assessed in the present study. Leveraging a multi-omics approach may reveal other SBAs that have proportionally different representation when there is diminished 7α-dehydroxylation capability by the gut microbiota.

The overall sample size represents a limitation that can be commonly encountered in clinical veterinary studies. Still, the sample size presented here represents an expansion on the previous investigation of fecal bile acid concentrations in CKD cats (Hall et al., 2020) and is one of only a few investigations to employ multi-omics by pairing assessment of the gut microbiome and targeted fecal bile acid composition in cats (Rowe and Winston, 2024). As previously discussed, the lack of strict dietary controls is also limiting along with the propensity of cats to develop histopathologic evidence of significant gastrointestinal disease in the absence of clinical signs (Marsilio et al., 2019). However, it is important to note that these limitations also represent a realistic feline population that would be encountered in clinical practice. Additionally, the availability of only forward reads from SRA is an inherent limitation of the *post-hoc* analysis presented here compared to the increased certainty of sequenced nucleotides from paired-end sequencing. However, as previously mentioned microbiome results from the previous publication (Summers et al., 2019) were replicated with the updated amplicon sequencing pipelined used herein.

Overall, the finding of reduced fecal concentrations of the SBA UDCA in cats with CKD is significant given the known ability of UDCA to modulate inflammation and fibrosis, including in rodent models of kidney injury (Yang et al., 2020; Abd-Elhamid et al., 2018; Brevini et al., 2023). Translational investigation of this finding warrants further exploration of fecal bile acids in people with CKD. Given these results, we postulate that commercially available and FDA-approved in people Ursodiol (ursodeoxycholic acid) could be considered for study as a component of multimodal treatment in human and veterinary patients with CKD. Separate from UDCA, fecal bile acid dysmetabolism characterized by <50% SBAs also occurs in a subpopulation of cats with CKD. The dysmetabolism is partially explained by reduced abundance of *P. hiranonis*, which to date is the only described member of the feline gut microbiota with the capability to perform 7α-dehydroxylation. Members of the order *Oscillospirales* displayed a similar pattern in correlation of abundance of SBAs within the bile acid pool. Metagenomic characterization of *Oscillospirales* have only recently suggested a role in bile acid metabolism, including the genetic potential to perform 7α-dehydroxylation in the human gut microbiota (Vital et al., 2019; Kim et al., 2022), making application of metagenomics an important next step to better characterize the phenomenon first described here in cats. If these microbial community members are shown to have the same genetic potential as described in the human gut microbes, it strengthens the use of cats with CKD as a potential translational spontaneously occurring disease model to explore the role of bile acid metabolism in the gut-kidney axis.

## Data Availability

The datasets presented in this study can be found in online repositories. The names of the repository/repositories and accession number(s) can be found at: https://www.ncbi.nlm.nih.gov/, SRP117611.

## References

[ref1] Abd-Elhamid T. H. Elgamal D. A. Ali S. S. Ali F. E. M. Hassanein E. H. M. El-Shoura E. A. M.. (2018). Reno-protective effects of ursodeoxycholic acid against gentamicin-induced nephrotoxicity through modulation of NF-κB, eNOS and caspase-3 expressions. Cell Tissue Res. 374, 367–387. doi: 10.1007/s00441-018-2886-y, PMID: 30078101

[ref2] Altschul S. F. Gish W. Miller W. Myers E. W. Lipman D. J. (1990). Basic local alignment search tool. J. Mol. Biol. 215, 403–410. doi: 10.1016/S0022-2836(05)80360-2, PMID: 2231712

[ref3] Anantharaman-Barr G. Ballèvre O. Gicquello P. Bracco-Hammer I. Vuichoud J. Montigon F.. (1994). Fecal bile acid excretion and taurine status in cats fed canned and dry diets. J. Nutr. 124, 2546S–2551S. 7996234 10.1093/jn/124.suppl_12.2546S

[ref4] Bartges J. W. (2012). Chronic kidney disease in dogs and cats. Vet. Clin. 42, 669–92, vi. doi: 10.1016/j.cvsm.2012.04.008, PMID: 22720808

[ref5] Benjamini Y. Krieger A. M. Yekutieli D. (2006). Adaptive linear step-up procedures that control the false discovery rate. Biometrika 93, 491–507. doi: 10.1093/biomet/93.3.491, PMID: 27051004

[ref6] Brevini T. Maes M. Webb G. J. John B. V. Fuchs C. D. Buescher G.. (2023). FXR inhibition may protect from SARS-CoV-2 infection by reducing ACE2. Nature 615, 134–142. doi: 10.1038/s41586-022-05594-0, PMID: 36470304 PMC9977684

[ref7] Callahan B. J. McMurdie P. J. Rosen M. J. Han A. W. Johnson A. J. A. Holmes S. P. (2016). DADA2: high-resolution sample inference from Illumina amplicon data. Nat. Methods 13, 581–583. doi: 10.1038/nmeth.3869, PMID: 27214047 PMC4927377

[ref8] Chávez-Talavera O. Tailleux A. Lefebvre P. Staels B. (2017). Bile acid control of metabolism and inflammation in obesity, type 2 diabetes, dyslipidemia, and nonalcoholic fatty liver disease. Gastroenterology 152, 1679–1694.e3. doi: 10.1053/j.gastro.2017.01.055, PMID: 28214524

[ref9] Chen Y. Y. Chen D. Q. Chen L. Liu J. R. Vaziri N. D. Guo Y.. (2019). Microbiome–metabolome reveals the contribution of gut–kidney axis on kidney disease. J. Transl. Med. 17:5. doi: 10.1186/s12967-018-1756-4, PMID: 30602367 PMC6317198

[ref10] Ciaula A. D. Garruti G. Baccetto R. L. Molina-Molina E. Bonfrate L. Portincasa P.. (2018). Bile acid physiology. Ann. Hepatol. 16, 4–14. doi: 10.5604/01.3001.0010.5493 29080336

[ref11] Clarke K. R. (1993). Non-parametric multivariate analyses of changes in community structure. Aust. J. Ecol. 18, 117–143. doi: 10.1111/j.1442-9993.1993.tb00438.x, PMID: 35313505

[ref12] Cline M. G. Burns K. M. Coe J. B. Downing R. Durzi T. Murphy M.. (2021). 2021 AAHA nutrition and weight management guidelines for dogs and cats*. J. Am. Anim. Hosp. Assoc. 57, 153–178. doi: 10.5326/JAAHA-MS-7232, PMID: 34228790

[ref13] Coleman J. P. Hudson L. L. Adams M. J. (1994). Characterization and regulation of the NADP-linked 7 alpha-hydroxysteroid dehydrogenase gene from *Clostridium sordellii*. J. Bacteriol. 176, 4865–4874. doi: 10.1128/jb.176.16.4865-4874.1994, PMID: 8050999 PMC196321

[ref14] Collins S. L. Stine J. G. Bisanz J. E. Okafor C. D. Patterson A. D. (2023). Bile acids and the gut microbiota: metabolic interactions and impacts on disease. Nat. Rev. Microbiol. 21, 236–247. doi: 10.1038/s41579-022-00805-x, PMID: 36253479 PMC12536349

[ref15] Ephraim E. Jewell D. E. (2021). Effect of nutrition on age-related metabolic markers and the gut microbiota in cats. Microorganisms 9:2430. doi: 10.3390/microorganisms9122430, PMID: 34946032 PMC8706506

[ref16] Foley M. H. O’Flaherty S. Barrangou R. Theriot C. M. (2019). Bile salt hydrolases: gatekeepers of bile acid metabolism and host-microbiome crosstalk in the gastrointestinal tract. PLoS Pathog. 15:e1007581. doi: 10.1371/journal.ppat.1007581, PMID: 30845232 PMC6405046

[ref17] Funabashi M. Grove T. L. Wang M. Varma Y. McFadden M. E. Brown L. C.. (2020). A metabolic pathway for bile acid dehydroxylation by the gut microbiome. Nature 582, 566–570. doi: 10.1038/s41586-020-2396-4, PMID: 32555455 PMC7319900

[ref18] GitHub (2023) Downloading SRA toolkit. Available at: https://github.com/ncbi/sra-tools/wiki/01.-Downloading-SRA-Toolkit (Accessed November 5, 2023).

[ref19] Goulis J. Leandro G. Burroughs A. K. (1999). Randomised controlled trials of ursodeoxycholic-acid therapy for primary biliary cirrhosis: a meta-analysis. Lancet 354, 1053–1060. doi: 10.1016/S0140-6736(98)11293-X, PMID: 10509495

[ref20] Hall J. A. Jewell D. E. Ephraim E. (2020). Changes in the fecal metabolome are associated with feeding Fiber not health status in cats with chronic kidney disease. Meta 10:281. doi: 10.3390/metabo10070281, PMID: 32660033 PMC7407581

[ref21] Herman-Edelstein M. Weinstein T. Levi M. (2018). Bile acid receptors and the kidney. Curr. Opin. Nephrol. Hypertens. 27, 56–62. doi: 10.1097/MNH.0000000000000374, PMID: 29045336

[ref22] IRIS Kidney (2023) Guidelines - IRIS Staging of CKD [Internet]. Available at: http://www.iris-kidney.com/guidelines/staging.html (Accessed November 5, 2023).

[ref23] Jackson M. I. Waldy C. Jewell D. E. (2020). Dietary resistant starch preserved through mild extrusion of grain alters fecal microbiome metabolism of dietary macronutrients while increasing immunoglobulin a in the cat. PLoS One 15:e0241037. doi: 10.1371/journal.pone.0241037, PMID: 33141838 PMC7608938

[ref24] Jepson R. E. (2016). Current understanding of the pathogenesis of progressive chronic kidney disease in cats. Vet. Clin. N. Am. Small Anim. Pract. 46, 1015–1048. doi: 10.1016/j.cvsm.2016.06.002, PMID: 27461408

[ref25] Kieler I. N. Mølbak L. Hansen L. L. Hermann-Bank ML Bjornvad C. R. (2016). Overweight and the feline gut microbiome – a pilot study. J. Anim. Physiol. Anim. Nutr. 100, 478–484. doi: 10.1111/jpn.12409, PMID: 26452635

[ref26] Kim K. H. Park D. Jia B. Baek J. H. Hahn Y. Jeon C. O. (2022). Identification and characterization of major bile acid 7α-Dehydroxylating Bacteria in the human gut. mSystems. 7, e00455–e00422. doi: 10.1128/msystems.00455-22 35736002 PMC9426597

[ref27] Kitahara M. Takamine F. Imamura T. Benno Y. (2000). VPI 12708 and related strains with high bile acid 7alpha-dehydroxylating activity to Clostridium scindens and proposal of *Clostridium hylemonae* sp. nov., isolated from human faeces. Int. J. Syst. Evol. Microbiol. 50, 971–978. doi: 10.1099/00207713-50-3-971, PMID: 10843034

[ref28] Kitahara M. Takamine F. Imamura T. Benno Y. (2001). *Clostridium hiranonis* sp. nov., a human intestinal bacterium with bile acid 7alpha-dehydroxylating activity. Int. J. Syst. Evol. Microbiol. 51, 39–44. doi: 10.1099/00207713-51-1-39 11211270

[ref29] Lavelle A. Nancey S. Reimund J. M. Laharie D. Marteau P. Treton X.. (2022). Fecal microbiota and bile acids in IBD patients undergoing screening for colorectal cancer. Gut Microbes 14:2078620. doi: 10.1080/19490976.2022.2078620, PMID: 35638103 PMC9176255

[ref30] Lee J. W. Cowley E. S. Wolf P. G. Doden H. L. Murai T. Caicedo K. Y. O.. (2022). Formation of secondary Allo-bile acids by novel enzymes from gut Firmicutes. Gut Microbes 14:2132903. doi: 10.1080/19490976.2022.2132903, PMID: 36343662 PMC9645264

[ref31] Li R. Zeng L. Xie S. Chen J. Yu Y. Zhong L. (2019). Targeted metabolomics study of serum bile acid profile in patients with end-stage renal disease undergoing hemodialysis. PeerJ. 7:e7145. doi: 10.7717/peerj.8265, PMID: 31245185 PMC6585905

[ref32] Lloyd-Price J. Arze C. Ananthakrishnan A. N. Schirmer M. Avila-Pacheco J. Poon T. W.. (2019). Multi-omics of the gut microbial ecosystem in inflammatory bowel diseases. Nature 569, 655–662. doi: 10.1038/s41586-019-1237-9, PMID: 31142855 PMC6650278

[ref33] Lou D. Wang B. Tan J. Zhu L. Cen X. Ji Q.. (2016). The three-dimensional structure of *Clostridium absonum* 7α-hydroxysteroid dehydrogenase: new insights into the conserved arginines for NADP(H) recognition. Sci. Rep. 6:22885. doi: 10.1038/srep22885 26961171 PMC4785404

[ref34] Love M. I. Huber W. Anders S. (2014). Moderated estimation of fold change and dispersion for RNA-seq data with DESeq2. Genome Biol. 15:550. doi: 10.1186/s13059-014-0550-8, PMID: 25516281 PMC4302049

[ref35] Lucas L. N. Barrett K. Kerby R. L. Zhang Q. Cattaneo L. E. Stevenson D.. (2021). Dominant bacterial Phyla from the human gut show widespread ability to transform and conjugate bile acids. mSystems. 6:21. doi: 10.1128/msystems.00805-21, PMID: 34463573 PMC12338150

[ref36] Ma X. Brinker E. Graff E. C. Cao W. Gross A. L. Johnson A. K.. (2022). Whole-genome shotgun metagenomic sequencing reveals distinct gut microbiome signatures of obese cats. Microbiol. Spectr. 10, e00837–e00822. doi: 10.1128/spectrum.00837-22 35467389 PMC9241680

[ref37] Marino C. L. Lascelles B. D. X. Vaden S. L. Gruen M. E. Marks S. L. (2014). Prevalence and classification of chronic kidney disease in cats randomly selected from four age groups and in cats recruited for degenerative joint disease studies. J. Feline Med. Surg. 16, 465–472. doi: 10.1177/1098612X13511446, PMID: 24217707 PMC4414065

[ref38] Marsilio S. Ackermann M. R. Lidbury J. A. Suchodolski J. S. Steiner J. M. (2019). Results of histopathology, immunohistochemistry, and molecular clonality testing of small intestinal biopsy specimens from clinically healthy client-owned cats. J. Vet. Intern. Med. 33, 551–558. doi: 10.1111/jvim.15455, PMID: 30820999 PMC6430868

[ref39] Martin M. (2011). Cutadapt removes adapter sequences from high-throughput sequencing reads. EMBnet. J. 17, 10–12. doi: 10.14806/ej.17.1.200, PMID: 28715235

[ref40] McLeland S. M. Cianciolo R. E. Duncan C. G. Quimby J. M. (2015). A comparison of biochemical and histopathologic staging in cats with chronic kidney disease. Vet. Pathol. 52, 524–534. doi: 10.1177/0300985814561095, PMID: 25516066

[ref41] McMillin M. DeMorrow S. (2016). Effects of bile acids on neurological function and disease. FASEB J. 30, 3658–3668. doi: 10.1096/fj.201600275R, PMID: 27468758 PMC5067249

[ref42] McMurdie P. J. Holmes S. (2013). Phyloseq: an R package for reproducible interactive analysis and graphics of microbiome census data. PLoS One 8:e61217. doi: 10.1371/journal.pone.0061217, PMID: 23630581 PMC3632530

[ref43] Nealon N. J. Wood A. Rudinsky A. J. Klein H. Salerno M. Parker V. J.. (2023). Fecal identification markers impact the feline fecal microbiota. Front Vet Sci. 10:1039931. doi: 10.3389/fvets.2023.1039931 36846255 PMC9946173

[ref44] Oksanen J. Simpson G. L. Blanchet F. G. Kindt R. Legendre P. Minchin P. R.. (2022) Vegan: community ecology package [internet]. Available at: https://CRAN.R-project.org/package=vegan (Accessed April 3, 2023).

[ref45] Otte C. M. A. Penning L. C. Rothuizen J. Favier R. P. (2013). Retrospective comparison of prednisolone and ursodeoxycholic acid for the treatment of feline lymphocytic cholangitis. Vet. J. 195, 205–209. doi: 10.1016/j.tvjl.2012.06.020, PMID: 22840210

[ref46] Perino A. Schoonjans K. (2022). Metabolic messengers: bile acids. Nat. Metab. 4, 416–423. doi: 10.1038/s42255-022-00559-z, PMID: 35338368

[ref47] Pilla R. Suchodolski J. S. (2021). The gut microbiome of dogs and cats, and the influence of diet. Vet. Clin. 51, 605–621. doi: 10.1016/j.cvsm.2021.01.002, PMID: 33653538

[ref48] Pinart M. Dötsch A. Schlicht K. Laudes M. Bouwman J. Forslund S. K.. (2022). Gut microbiome composition in obese and non-obese persons: a systematic review and Meta-analysis. Nutrients 14:12. doi: 10.3390/nu14010012 PMC8746372 35010887

[ref49] Poupon R. (2012). Ursodeoxycholic acid and bile-acid mimetics as therapeutic agents for cholestatic liver diseases: an overview of their mechanisms of action. Clin. Res. Hepatol. Gastroenterol. 36, S3–S12. doi: 10.1016/S2210-7401(12)70015-3, PMID: 23141891

[ref50] Poupon R. E. Balkau B. Eschwège E. Poupon R. (1991). A multicenter, controlled trial of Ursodiol for the treatment of primary biliary cirrhosis. N. Engl. J. Med. 324, 1548–1554. doi: 10.1056/NEJM199105303242204, PMID: 1674105

[ref51] Quast C. Pruesse E. Yilmaz P. Gerken J. Schweer T. Yarza P.. (2013). The SILVA ribosomal RNA gene database project: improved data processing and web-based tools. Nucleic Acids Res. 41, D590–D596. doi: 10.1093/nar/gks1219, PMID: 23193283 PMC3531112

[ref52] R Core Team (2013). R: A language and environment for statistical Computing. Vienna: R Core Team.

[ref53] Ridlon J. M. Daniel S. L. Gaskins H. R. (2023). The Hylemon-Björkhem pathway of bile acid 7-dehydroxylation: history, biochemistry, and microbiology. J. Lipid Res. 64:100392. doi: 10.1016/j.jlr.2023.100392, PMID: 37211250 PMC10382948

[ref54] Ridlon J. M. Kang D. J. Hylemon P. B. (2006). Bile salt biotransformations by human intestinal bacteria. J. Lipid Res. 47, 241–259. doi: 10.1194/jlr.R500013-JLR200, PMID: 16299351

[ref55] Ridlon J. M. Kang D. J. Hylemon P. B. (2010). Isolation and characterization of a bile acid inducible 7α-dehydroxylating operon in *Clostridium hylemonae* TN271. Anaerobe 16, 137–146. doi: 10.1016/j.anaerobe.2009.05.004, PMID: 19464381 PMC6262846

[ref56] Rodríguez-Morató J. Matthan N. R. (2020). Nutrition and gastrointestinal microbiota, microbial-derived secondary bile acids, and cardiovascular disease. Curr. Atheroscler. Rep. 22:47. doi: 10.1007/s11883-020-00863-7, PMID: 32681421

[ref57] Rowe J. C. Winston J. A. (2024). Collaborative metabolism: gut microbes play a key role in canine and feline bile acid metabolism. Vet. Sci. 11:94. doi: 10.3390/vetsci11020094, PMID: 38393112 PMC10892723

[ref58] Rowe J. C. Winston J. A. Parker V. J. McCool K. E. Suchodolski J. S. Lopes R.. (2024). Gut microbiota promoting propionic acid production accompanies caloric restriction-induced intentional weight loss in cats. Sci. Rep. 14:11901. doi: 10.1038/s41598-024-62243-4, PMID: 38789518 PMC11126632

[ref59] Summers S. C. Quimby J. M. Isaiah A. Suchodolski J. S. Lunghofer P. J. Gustafson D. L. (2019). The fecal microbiome and serum concentrations of indoxyl sulfate and p-cresol sulfate in cats with chronic kidney disease. J. Vet. Intern. Med. 33, 662–669. doi: 10.1111/jvim.15389, PMID: 30561098 PMC6430892

[ref60] Stavroulaki E. M. Suchodolski J. S. Pilla R. Fosgate G. T. Sung C. H. Lidbury J.. (2022). The serum and fecal Metabolomic profiles of growing kittens treated with amoxicillin/clavulanic acid or doxycycline. Animals 12:330. doi: 10.3390/ani12030330, PMID: 35158655 PMC8833518

[ref61] Streidl T. Karkossa I. Segura Muñoz R. R. Eberl C. Zaufel A. Plagge J.. (2021). The gut bacterium Extibacter muris produces secondary bile acids and influences liver physiology in gnotobiotic mice. Gut Microbes 13:1854008. doi: 10.1080/19490976.2020.1854008 PMC7781625 33382950

[ref62] Sung C. H. Marsilio S. Chow B. Zornow K. A. Slovak J. E. Pilla R.. (2022). Dysbiosis index to evaluate the fecal microbiota in healthy cats and cats with chronic enteropathies. J. Feline Med. Surg. 24, e1–e12. doi: 10.1177/1098612X221077876, PMID: 35266809 PMC9160961

[ref63] Sung C. H. Pilla R. Marsilio S. Chow B. Zornow K. A. Slovak J. E.. (2023). Fecal concentrations of long-chain fatty acids, sterols, and unconjugated bile acids in cats with chronic enteropathy. Animals 13:2753. doi: 10.3390/ani13172753, PMID: 37685017 PMC10486672

[ref64] Sutherland J. D. Williams C. N. (1985). Bile acid induction of 7 alpha- and 7 beta-hydroxysteroid dehydrogenases in *Clostridium limosum*. J. Lipid Res. 26, 344–350. doi: 10.1016/S0022-2275(20)34377-7, PMID: 3857290

[ref65] Ticho A. L. Malhotra P. Dudeja P. K. Gill R. K. Alrefai W. A. (2019). Bile acid receptors and gastrointestinal functions. Liver Res. 3, 31–39. doi: 10.1016/j.livres.2019.01.001, PMID: 32368358 PMC7197881

[ref66] Vital M. Rud T. Rath S. Pieper D. H. Schlüter D. (2019). Diversity of Bacteria exhibiting bile acid-inducible 7α-dehydroxylation genes in the human gut. Comput. Struct. Biotechnol. J. 17, 1016–1019. doi: 10.1016/j.csbj.2019.07.012, PMID: 31428294 PMC6692061

[ref67] Wang X. X. Edelstein M. H. Gafter U. Qiu L. Luo Y. Dobrinskikh E.. (2016). G protein-coupled bile acid receptor TGR5 activation inhibits kidney disease in obesity and diabetes. J. Am. Soc. Nephrol. 27, 1362–1378. doi: 10.1681/ASN.2014121271, PMID: 26424786 PMC4849814

[ref68] Ward J. B. J. Lajczak N. K. Kelly O. B. O’Dwyer A. M. Giddam A. K. Ní Gabhann J.. (2017). Ursodeoxycholic acid and lithocholic acid exert anti-inflammatory actions in the colon. American journal of physiology-gastrointestinal and liver. Physiology 312, G550–G558. doi: 10.1152/ajpgi.00256.2016 28360029

[ref69] Whittemore J. C. Stokes J. E. Laia N. L. Price J. M. Suchodolski J. S. (2018). Short and long-term effects of a synbiotic on clinical signs, the fecal microbiome, and metabolomic profiles in healthy research cats receiving clindamycin: a randomized, controlled trial. PeerJ. 6:e5130. doi: 10.7717/peerj.5130, PMID: 30038854 PMC6054061

[ref70] Whittemore J. C. Stokes J. E. Price J. M. Suchodolski J. S. (2019). Effects of a synbiotic on the fecal microbiome and metabolomic profiles of healthy research cats administered clindamycin: a randomized, controlled trial. Gut Microbes 10, 521–539. doi: 10.1080/19490976.2018.1560754, PMID: 30709324 PMC6748608

[ref71] Winston J. A. Theriot C. M. (2016). Impact of microbial derived secondary bile acids on colonization resistance against *Clostridium difficile* in the gastrointestinal tract. Anaerobe 41, 44–50. doi: 10.1016/j.anaerobe.2016.05.003, PMID: 27163871 PMC5050083

[ref72] Winston J. A. Theriot C. M. (2020). Diversification of host bile acids by members of the gut microbiota. Gut Microbes 11, 158–171. doi: 10.1080/19490976.2019.1674124, PMID: 31595814 PMC7053883

[ref73] Yang Y. Liu S. Gao H. Wang P. Zhang Y. Zhang A.. (2020). Ursodeoxycholic acid protects against cisplatin-induced acute kidney injury and mitochondrial dysfunction through acting on ALDH1L2. Free Radic. Biol. Med. 152, 821–837. doi: 10.1016/j.freeradbiomed.2020.01.182, PMID: 32004633

[ref74] Ye H. L. Zhang J. W. Chen X. Z. Wu P. B. Chen L. Zhang G. (2020). Ursodeoxycholic acid alleviates experimental liver fibrosis involving inhibition of autophagy. Life Sci. 242:117175. doi: 10.1016/j.lfs.2019.117175, PMID: 31843528

[ref75] Zhou W. Wu W. H. Si Z. L. Liu H. L. Wang H. Jiang H.. (2022). The gut microbe *Bacteroides fragilis* ameliorates renal fibrosis in mice. Nat. Commun. 13:6081. doi: 10.1038/s41467-022-35690-8, PMID: 36241632 PMC9568537

