# Supplementary material for: Fecal bile acid dysmetabolism and reduced ursodeoxycholic acid correlate with novel microbial signatures in feline chronic kidney disease
Source: Front Microbiol. 2024 Oct 21;15:1458090. doi: 10.3389/fmicb.2024.1458090 (PMC11532117; doi:10.3389/fmicb.2024.1458090)
Supplement: Supplementary file 1 [file Table_1.DOCX]

Supplementary Material

|  | Healthy (n = 6) | IRIS 2 CKD (n = 17) | IRIS 3+4 CKD (n = 11) |
| --- | --- | --- | --- |
| Age (years) | 10 (8.5-11.5) | 15 (7-22) | 15 (7-17) |
| BCS | 5 (4-5) | 5 (3-7) | 5 (2-7) |
| Creatinine (mg/dL) | 1.2 (0.9-1.4) | 2.0 (1.6-2.6) | 3.4 (2.9-6.9) |
| BUN (mg/dL) | 21.5 (18-24) | 43 (20-60) | 52 (33-98) |
| Hematocrit (%) | 42 (38-46) | 36 (31-46) | 35 (24-39) |

**Supplementary Table 1.** Basic demographic information for all client-owned cats utilized in the study. Data within table are shown as median with range in parentheses. BCS = body condition score. BUN = blood urea nitrogen.
